# Allosteric mechanism of the circadian protein Vivid resolved through Markov state model and machine learning analysis

**DOI:** 10.1371/journal.pcbi.1006801

**Published:** 2019-02-19

**Authors:** Hongyu Zhou, Zheng Dong, Gennady Verkhivker, Brian D. Zoltowski, Peng Tao

**Affiliations:** 1 Department of Chemistry, Center for Scientific Computation, Center for Drug Discovery, Design, and Delivery (CD4), Southern Methodist University, Dallas, Texas, United States of America; 2 Graduate Program in Computational and Data Sciences, Schmid College of Science and Technology, Chapman University, Orange, California, United States of America; 3 Chapman University School of Pharmacy, Irvine, California, United States of America; University of Maryland School of Pharmacy, UNITED STATES

## Abstract

The fungal circadian clock photoreceptor Vivid (VVD) contains a photosensitive allosteric light, oxygen, voltage (LOV) domain that undergoes a large N-terminal conformational change. The mechanism by which a blue-light driven covalent bond formation leads to a global conformational change remains unclear, which hinders the further development of VVD as an optogenetic tool. We answered this question through a novel computational platform integrating Markov state models, machine learning methods, and newly developed community analysis algorithms. Applying this new integrative approach, we provided a quantitative evaluation of the contribution from the covalent bond to the protein global conformational change, and proposed an atomistic allosteric mechanism leading to the discovery of the unexpected importance of A’α/Aβ and previously overlooked Eα/Fα loops in the conformational change. This approach could be applicable to other allosteric proteins in general to provide interpretable atomistic representations of their otherwise elusive allosteric mechanisms.

## Author summary

Allostery is an important but elusive property that governs critical functionality of many proteins. Quantitative analysis is needed to provide significant insight into protein allostery and lead to better prediction power of this ubiquitous phenomenon. We developed machine learning methods based on robust Markov state model to delineate allosteric mechanism of Vivid as an allosteric protein in the filamentous fungus *Neurospora crassa*, regulating circadian rhythm of this organism. We accurately reconstructed the equilibrium distributions for two allosteric configurations of Vivid, and determined structural differences among these states. Intriguingly, the novel community analysis derived from machine learning methods reveals the importance of two loop regions for Vivid allostery through quantitative evaluations with statistical significance.

## Introduction

Light, oxygen, or voltage (LOV) domains are small, commutable elements that couple blue-light activation to protein conformational changes for blue-light responses in bacteria, archaea, fungi and plants. One common feature shared by all LOV domains is that a cofactor, flavin adenine dinucleotide (FAD), flavin mononucleotide (FMN) or riboflavin,[1] forms a covalent bond with a conserved cysteine residue upon external light activation. Covalent adduct formation, subsequently facilitates a large conformational change in the protein leading to alteration in enzyme activity and/or protein-protein interactions.[2, 3] The mechanism of LOV domain global conformational change induced by covalent bond is widely accepted as an allosteric process and remains as a focal point of LOV domain studies with an aim of developing novel optogenetic tool through manipulating LOV domain allostery.

Vivid (VVD) is a LOV-domain containing photoreceptor from the filamentous fungus *Neurospora crassa* that modulates circadian rhythms in this organism. In *Neurospora*, circadian-clock regulated gene expression is dictated by a heterodimeric complex involving the photosensitive protein White Collar-1 (WC-1) and the non-photosensitive protein WC-2. Upon blue-light exposure it is believed that an additional copy of WC-1 is recruited to light-responsive elements (LRE’s) to form a hetero-trimeric complex involving a WC-1 homodimer (WCC). One of the blue-light induced gene products is VVD, which competes for binding to WC-1 to disrupt the WCC and modulate light-induced gene expression.[4] VVD activity is dependent upon activation through blue light, where Cys108 of VVD forms a covalent bond between its sulfur and C4a position of co-factor FAD, which in-turn induces N terminus conformational change of VVD necessary for WCC regulation. Based on previous experimental and computational studies, VVD serves as a good candidate for optogenetic tool developing. However the mechanism of VVD allostery correlated with its global conformational change upon covalent bond formation is still an open question.

Investigating protein allostery through computational means is effective and is under constant development. Various studies were conducted to reveal the underlining mechanisms for different allosteric proteins.[5–7] One method developed, named rigid residue scan (RRS), has been applied in several systems including PDZ3[8] and PDZ2 domains.[9] Other methods including dynamical network analysis[10], elastic network model[11], relative entropy based allostery network[12], sequence and structural analysis[13], correlation based analysis[14] etc. have been widely applied on many allosteric systems. Markov state model (MSM) of molecular kinetics has been widely used in recent years to estimate long-time kinetic information from short trajectories.[15–17] Molecular dynamics (MD) simulations often involve long time samplings or enhanced sampling to detect rare events with statistical significance.[17] Implementing predefined order parameters as reaction coordinates is a useful means to analyze protein simulations. However, the possibilities for neglecting the true kinetic information underlying the simulations with hidden important barriers remain as one intrinsic limitation of the predefined order parameters approaches.[18, 19] Although Markov state analysis could be applied to separate the structures based on their kinetic information, a quantitative strategy to measure the primary differences among different states is still absent. Long-time scale molecular dynamics simulations could provide sufficient sampling of the conformational landscape of proteins. But obtaining statistically significant insights into protein dynamics from massive simulation datasets presents a major challenge[20–22].

Artificial neural networks (ANN)[23, 24], tree based model including decision tree (DT)[25], and random forest (RT)[26] are widely applied as classification methods in machine learning. ANN mimics neural networks consisting of neurons in the brain, and has been applied in many classification problems to achieve high accuracy[27]. Decision tree was constructed to quantify the importance for making decisions or predictions of each dimension in the input data that is statistically relevant to relative entropy metrics in distinguishing between different distributions.[28–30] Recently, we demonstrated significant effectiveness of DT and ANN methods to build allostery classification models and identify allosterically important residues.[31, 32]

To gain further insight into VVD allosteric mechanism, more quantitative description would be necessary in addition to normal qualitative analysis of protein allostery. In this work, we developed a novel computational framework that can significantly boost the applicability of molecular simulation techniques to probe dynamic allostery in protein systems, and applied this approach on VVD. Specifically, we combined machine learning and dynamic community analysis of the residue interaction networks to obtain robust quantitative descriptions of conformational ensembles and protein states, and to rigorously correlate variations in conformational ensembles to underlying allosteric mechanisms. Both methods are enabled by a new application of machine learning and network modeling to the analysis of thermodynamic and kinetic information from MSM. The proposed models are applied to (a) rapidly recognize and identify structural and dynamic patterns of complex conformational ensembles; (b) identify key functional states that are hidden in the conformational ensembles, and (c) reconstruct the mechanisms of dynamics driven allostery through integration of machine learning and network analysis.

Using the proposed computational framework, we examined allosteric mechanisms of VVD and verified the impacts of the key local covalent bond upon photo excitation to global motions of VVD, and revealed the importance of A’α/Aβ and Eα/Fα loops in the conformational change. A good agreement between our analysis and experimental observations of VVD validated the applicability of the proposed approach, and provided structural insights into mechanism of conformational changes and allostery in allosteric proteins. Our methodology could facilitate the usage of VVD as an optogenetic tool by providing quantitative measurement of individual residues’ contribution to protein allostery.

## Results

### Markov states analysis of VVD simulation

**Fig 1:**
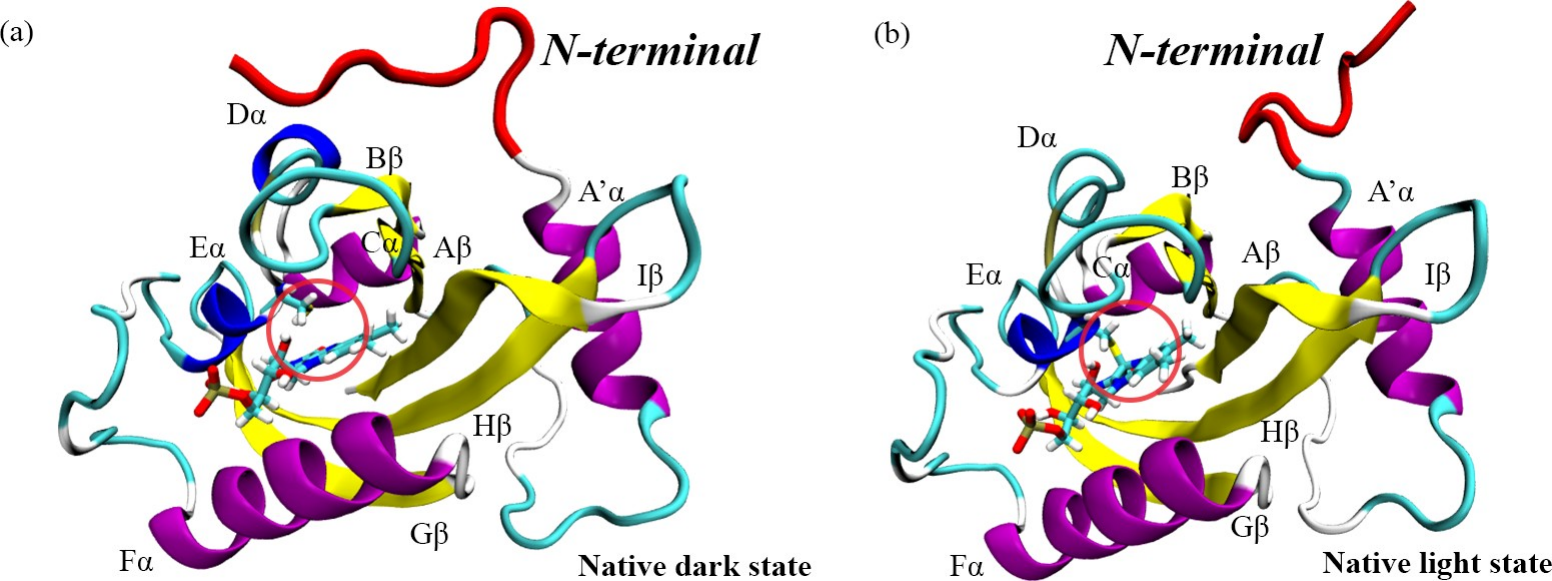
Two native states of VVD: (a) native dark state (PDB code: 2PD7) and (b) native light state (PDB code: 3RH8). The blue light could activate the dark state and lead to the formation of covalent bond circled in the native light state. The main conformational change lies in the N-terminal structure, which is highlighted in red.

**Fig 2:**
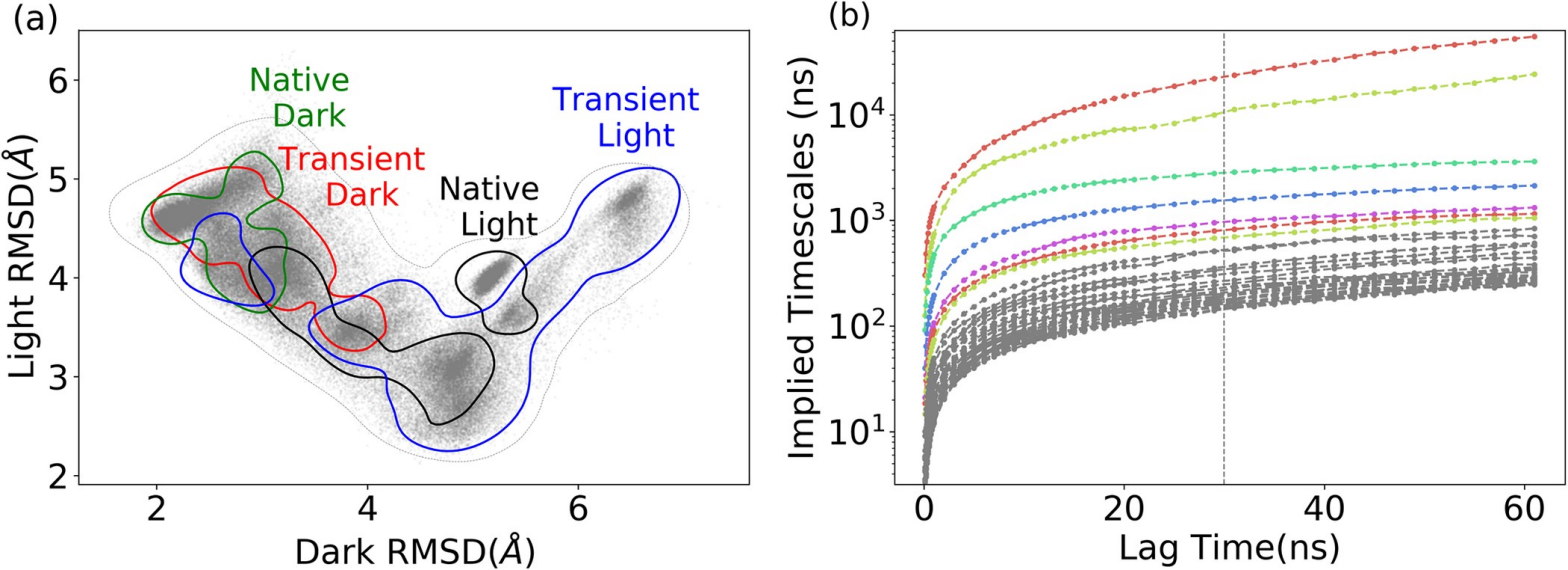
Molecular dynamics simulations of VVD protein with four different configurations, native dark and native light configurations, and transient dark and transient light configurations. (a) 2D-RMSD plot of the simulations with different configurations with reference to the native dark and light structures, respectively, (each contour in different color represents the samplings from three trajectories with the same configuration); (b) Estimated relaxation timescale based on transition probabilities among different microstates regarding with the different lag time as interval for analysis.

There are two native crystallographic structures of VVD: dark structure (without covalent bond formed between FAD and VVD residue Cys108) and light structure (with such covalent bond). We referred to these two states as native dark (non-bonded) and native light (bonded) configurations (Fig 1). To probe the response from protein with regard to the covalent bond between FAD and VVD, two new configurations were constructed: dark structure with the above covalent bond, and light state without the above covalent bond. We referred to these two states as transient dark (bonded) and transient light (non-bonded) configurations, respectively. Three independent 1 μs simulations were carried out for each configuration, leading to 12 μs production trajectories. All 12 μs trajectories of VVD are projected onto a two dimensional (2D) plot of root-mean-square deviation (RMSD) of VVD backbone alpha carbon atoms (Cα) with reference to the native dark and light structures, respectively (Fig 2A). The plot reveals that the simulations of light state configurations may reach the native dark state structure. On the contrary, the simulations of dark state configurations show less fluctuation than the light state configurations in simulations.

**Fig 3:**
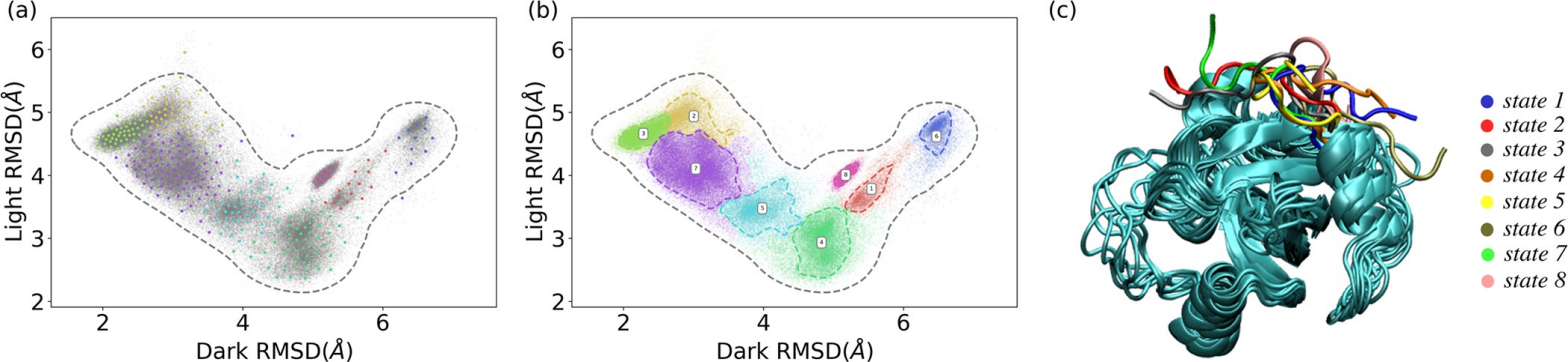
Markov state model (MSM) construction of eight macrostates based on the 300 microstates generated using *k*-means clustering analysis of VVD MD simulations. (a) Plot of 300 microstates on 2D RMSD surface belonging to eight macrostates with different colors representing different macrostates; (b) Distribution of each macrostate on the 2D RMSD surface; (c) Representative structures of eight macrostates.

In order to apply MSM analysis, *k*-means clustering analysis was applied to divide the sampling space into 300 microstates based on structural differences (Fig 3A). The transition probabilities were estimated among microstates at a specified interval of time named as lag time. An adequate lag time should be selected based on the convergence of the estimated relaxation timescale.[33] The data plotted in Fig 2B suggest that the estimated timescale is converged after 30 nanoseconds (dashed grey line), which is chosen as the lag time for the MSM analysis. The number of macrostates should be rigorously chosen to better represent the free energy landscape. Overall, having eight macrostates will result in the best separation to represent kinetically meaningful states on the free energy surface as shown in Fig 3A.

Perron-cluster cluster analysis (PCCA) was applied to map microstates onto macrostates based on the eigenfunction structure of transition probability matrix (Fig 3A and 3B). The representative structure for each macrostate is illustrated in Fig 3C. The averaged RMSDs of the macrostates 2, 3 and 7 with reference to the crystal dark and light structures of VVD are 2.84Å and 4.38Å, respectively. Similarly, the averaged RMSDs for the macrostates 1, 4, 5 and 8 are 4.69Å and 3.31Å with reference to the crystal dark and light structures, respectively. Locating at the top right corner of the 2D RMSD plot, macrostate 6 is far away from both crystal dark and light conformations of VVD, with the averaged RMSD values as 6.47Å and 4.62Å, respectively. As the sampling of macrostate 6 only occurs in transient light configuration simulations, the macrostate 6 is referred to as a “hidden” state, which cannot be reached in the simulation of native states.

To assess the effectiveness of the above analysis for VVD, we carried out time-structure independent components analysis (t-ICA) and principal component analysis (PCA) to verify that the markovian property is well maintained using MSM. For comparison purpose, 30ns as the lag time and total of eight macrostates are used for both t-ICA and PCA. The results for the comparison shown in S1(A)–S1(C) Fig represent the projection of VVD simulations onto the surfaces of 2D-RMSD, t-ICA and PCA, respectively. The relaxation timescales of these MSM models are shown in S1(E)–S1(G) Fig. The relaxation timescale estimated from t-ICA is significantly higher than the ones with 2D-RMSD and PCA, which indicates that t-ICA may capture slow kinetic components better than 2D-RMSD and PCA. However, the connectivity of microstates on the projection surface of t-ICA is lower than the one on 2D-RMSD or PCA surfaces. Thus the identified “strongest connected subgraph”[34] on the t-ICA surface does not contain all microstates. Based on the ergodic cutoff criterion, during the construction phase of MSM, 173 out of 300 microstates on t-ICA surface were discarded because they are weakly connected to other microstates. The highly disconnected communities indicate that the major t-ICs could be the spurious collective variables due to high dimensionality. Therefore, we selected top five features identified in Table 1 (which will be discussed later) and construct t-ICA surface only on those features. The projection surface is shown in S1(D) Fig and the relaxation timescale is shown in S1(H) Fig. Microstates grouped in eight macrostates on 2D-RMSD, t-ICA, PCA, and t-ICA with five features are illustrated in S1(I)–S1(L) Fig, respectively. With only five selected features, the t-ICA results are much improved, suggesting that t-ICA method works better with reduced dimensionality. The results of 2D-RMSD, PCA, and t-ICA with five selected features are similar with each other. However the axis of 2D-RMSD could better represent structural information than PCA or t-ICA with direct measurement of difference from the two key structures of VVD.

**Table 1:** Top 5 features in overall importance to distinguish difference states.

features	OVERALL	Rank: 1	Rank: 2	Rank: 3	Rank: 4
213 (T38 –G105)	2.83%	S3-S5: 4.28%	S1-S7: 4.03%	S5-S6: 4.00%	S2-S6: 4.00%
227 (T38 –K119)	2.38%	S2-S4: 4.55%	S3-S4: 4.00%	S1-S3: 4.00%	S2-S6: 4.00%
189 (T38 –K81)	1.75%	S3-S7: 4.28%	S2-S5: 4.08%	S3-S5: 4.05%	S1-S7: 4.02%
29 (H37 –V67)	1.52%	S1-S7: 4.05%	S3-S6: 4.00%	S2-S8: 3.99%	S3-S8: 3.99%
355 (L39 –E102)	1.51%	S5-S6: 5.72%	S4-S7: 5.48%	S1-S6: 5.31%	S4-S5: 4.44%

To ensure the kinetic similarity within the microstates during the clustering, the averaged RMSD in each microstate is plotted for 2D-RMSD, t-ICA, PCA and t-ICA with five selected features models in S2 Fig, respectively. It is assumed that the conformations with small RMSDs may interchange quickly. Practically, averaged RMSD inside each microstate smaller than 2.0Å is sufficient to imply the kinetic similarity within that microstate.[33] The averaged RMSDs are smaller than 2.0Å for all microstates using three models, indicating that the kinetic similarity within each microstate is well maintained. In addition, the markovian property using 2D-RMSD was also tested using Chapman-Kolmogorov test [17] by comparing the probability directly observed in the simulation with the estimated probability using lag time as 30ns (S3 Fig). To avoid spurious large error bar due to the difference of saving coordinates frequency between the reference study (0.2ps) and the current study (100ps), the denominator in the reference paper [17] equation 66 was replaced by the ratio of all transition count to the actual transition counts in the simulation. Therefore, the error bar is less dependent on the saving frequency of the simulation. The similarity between these two probabilities shown in S3 Fig suggests that the markovian property using 2D-RMSD as reaction coordinates for MSM model is well maintained.

**Fig 4:**
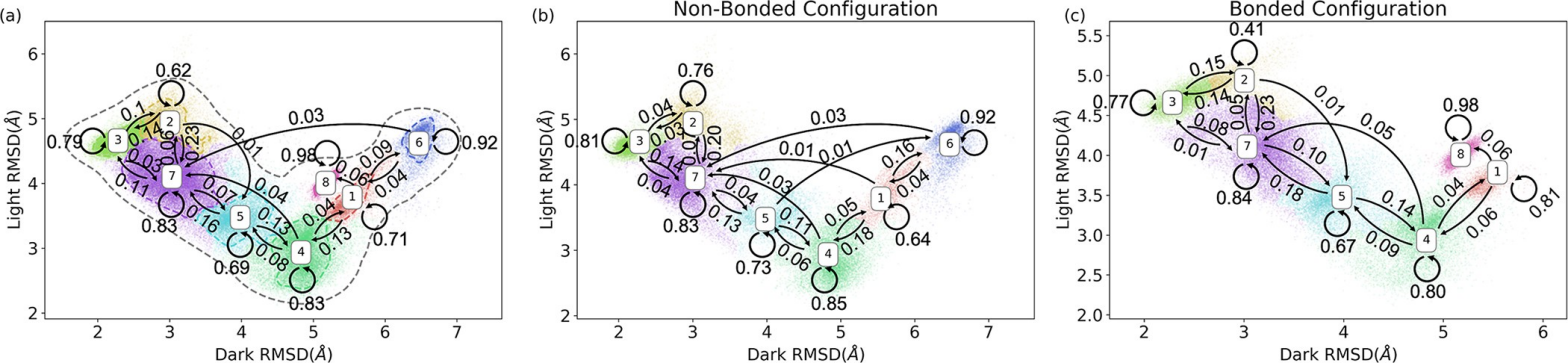
The transition probabilities estimated based on Markov state model among macrostates. (a) The transition probabilities estimated using all trajectories; (b) The transition probabilities estimated using non-bonded trajectories; (c) The transition probabilities estimated using bonded trajectories. State 6 only appears in the non-bonded configurations. State 8 only appears in the bonded configurations. The transition probability among macrostates are significantly different, showing that the formation of photo-induced covalent bond could mediate the kinetics of the system.

After the construction of MSM, the transition probabilities estimated among adjacent macrostates are shown in Fig 4. For each state, the probability to remain in the current state is higher than switching to other states, which suggests that each macrostate is a minimum on free energy surface, and the kinetic barriers prevent the switching to other states. The above transition probability matrix was calculated based on all 12 μs MD trajectories. To further explore the cofactor covalent bond effect, the transition probability matrices were calculated separately for six non-bonded (for native dark and transient light configurations) and six bonded trajectories (for native light and transient dark configurations). As shown in Fig 4B and 4C, forming of covalent bond has significant impact on the transition probabilities among macrostates, which suggests that the covalent bond could alter the free energy surface and energy barriers among different states.

**Fig 5:**
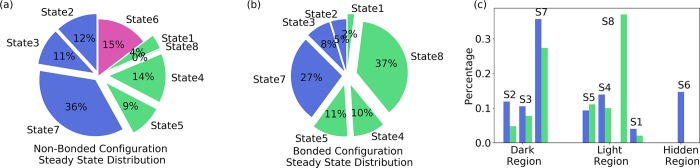
The estimated steady state distributions for each state based on Markov state model. (a) Steady state distribution based on non-bonded configuration simulations; (b) Steady state distribution based on bonded configuration simulations; (c) Steady state distribution differences between non-bonded and bonded configurations. States 2, 3, and 7 are considered as dark states region. States 1, 4, 5, and 8 are considered as light state region. State 6 is considered as hidden region.

The steady state distribution that the system may reach at the infinite time could be estimated based on the calculated transition probabilities. The eigenvector associated with the eigenvalue 1.0 for the transition probability matrix is the stationary distribution for each state. This is only an approximation, because after discretizing the phase space into microstates, the markovian properties may not hold precisely.[35] However, it is still valuable to investigate the distribution at infinite time (referred to as steady state thereafter) to obtain an overall picture regarding to the long time behavior. The steady state distributions based on the non-bonded (Fig 5A) and bonded trajectories (Fig 5B) are illustrated separately. For comparison purpose, the distributions based on non-bonded and bonded MD trajectories, which are referred to as ensemble distributions, are illustrated in S4 Fig. Overall, the steady state distribution differences between non-bonded and bonded configurations are significant (Fig 5C). The light state sampling is significantly enhanced in the bonded configuration, primarily in the state 8. In the non-bonded configuration, the samplings of dark state conformations including states 2, 3, and 7 are more extensive than the bonded configuration (Fig 5C). The hidden state (state 6) is only sampled in the bonded configurations. Similar with the Fig 4B and 4C comparison, these results indicate that the bonded configurations favor the native light state structure and the non-bonded configurations favor the native dark state structure. The convergence of simulations is verified by RMSD and configurational entropy in S5 Fig. The plot of configurational entropy (S5B Fig) indicates that the simulations are well converged after ~600ns samplings.

The sampled conformational space in the 12μs MD simulations has a remarkable agreement with our previous study of VVD through perturbed MD simulations.[36] Our analyses show that the MD simulations of transient light and dark configurations sampled larger conformational space than the native light and dark configurations. These results conform that the covalent bond could facilitate the conversion of dark state to the light state, agreeing with the experimental observations.[4, 37, 38] Markov state analysis provides more quantitative descriptions than intuitive interpretation based on sampling space. The covalent bond affects the transition probabilities among macrostates and the steady states/equilibrium distributions significantly. The steady states distribution can be considered as the free energy for each state. Therefore, the changes of steady states distribution can be regarded as the changes of free energy surface of the protein dynamics due to the formation of covalent bond. The transition probabilities between state3-state2, state7-state5, state5-state4 increase from (3%, 4%, 11%) to (14%, 10%, 14%), respectively, comparing the non-bonded and bonded configurations. The increase of the transition probability is significant, which lead to the estimated steady states distribution differences in dark and light states. These differences suggest that the transitions from dark state to light state could be triggered mainly by the formation of covalent bond without excitation energy dissipation.

Another difference is the behavior of conformations dwelling in state 1. Based on the non-bonded configuration simulations, starting from state 1, the probability for protein directly changing to state 8 is 0%, and to state 6 is 16%. In the bonded configuration simulations, these probabilities are 6% and 0%, respectively. Meanwhile, state 6 is regarded as a hidden state as it was not sampled in either native light or native dark configurations, and is structurally different from both crystal dark and light states. The different behavior of state 1 in non-bonded and bonded configurations could be interpreted as a stabilization effects to the light states from the covalent bond. With the covalent bond, the light state can be stabilized to the state 8 conformations without reaching hidden state 6.

Based on the above observations, we can hypothesize that the covalent bond has a significant role in light state conformation. With this covalent bond, the light state conformation could be stabilized. Otherwise it would be trapped in the hidden state conformations, which cannot be sampled in the native states. Our results demonstrated that even without energy available in an excited state upon blue light excitation, the single covalent bond could trigger the global conformational change of VVD.

The MSM analysis reveals the impacts of covalent bond between cofactor and protein in conformational distribution and free energy barriers among macrostates. However, the conformational characteristic for each state and the mechanism for the conformational changes are still unclear. Therefore, several supervised machine-learning models were applied to study the intrinsic structural properties of macrostates.

### Machine learning identifies key structural features and interactions that characterize allosterically important protein macrostates

**Fig 6:**
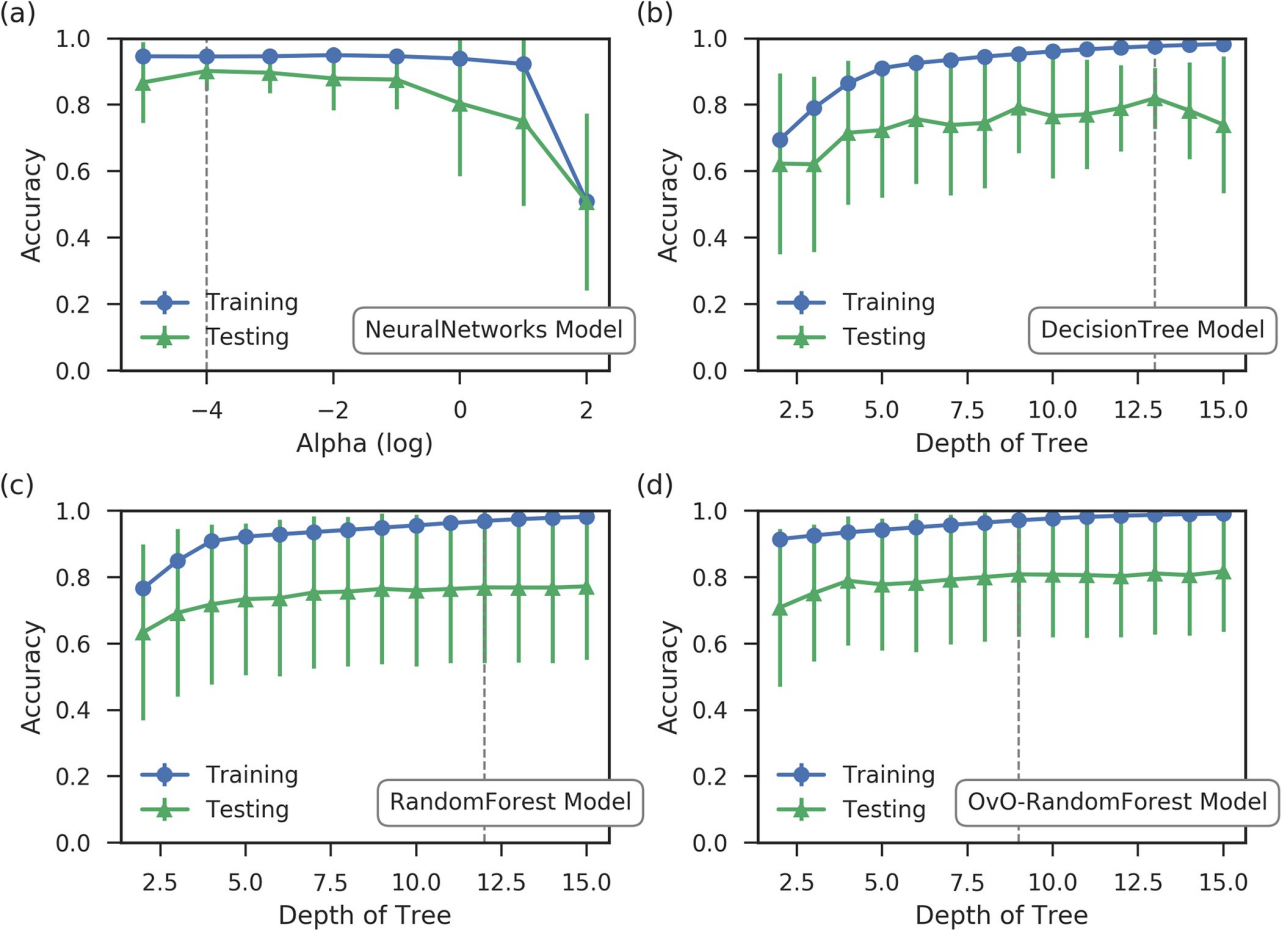
The training models for macrostates using different machine learning model: (a) Artificial neural networks, (b) decision tree, (c) random forest, and (d) one-vs-one random forest. All the training are based on 120,000 frames extracted from 12 μs MD trajectories. In each frame, 10,878 Cα pair-wise distances are used as features for model development. All models show high training and testing accuracy, validating the appropriation of machine learning models for VVD simulations.

To apply the supervised machine learning models and study the structural differences, appropriate collective variables are needed to describe a protein structure. For the small organic molecules, several descriptors including the topological torsion[39], reduced graph descriptor[40] have been developed, and widely used in quantitative structure–activity relationship (QSAR) and docking studies.[41, 42] Here, we chose the pair-wised distances of alpha carbon (Cα) of amino acids as translation and rotation invariant collective variables for protein structures in our simulations. Total 10,878 pair-wised distances were constructed based on 148 residues. For each simulation, frames are saved for every 100 picoseconds (ps), resulting in 10,000 frames for every 1 μs MD trajectory. Therefore, 120,000 “data points” with 10,878 features were extracted from 12 μs MD trajectories. Above macrostate analysis were used to label each frame. After the preparation of data, decision tree, random forest, one-vs-one random forest and artificial neural networks models were applied to distinguish the intrinsic conformational differences among macrostates. Dimensionality deduction was done by one-vs-one random forest before applying artificial neural networks model. Each machine learning model is described in Methodology section. The cross-validation was applied to refine the parameters of these models. The training and testing error of 12-fold cross-validation are plotted in Fig 6. The final selected parameters are indicated by dashed vertical line in each subplot.

**Fig 7:**
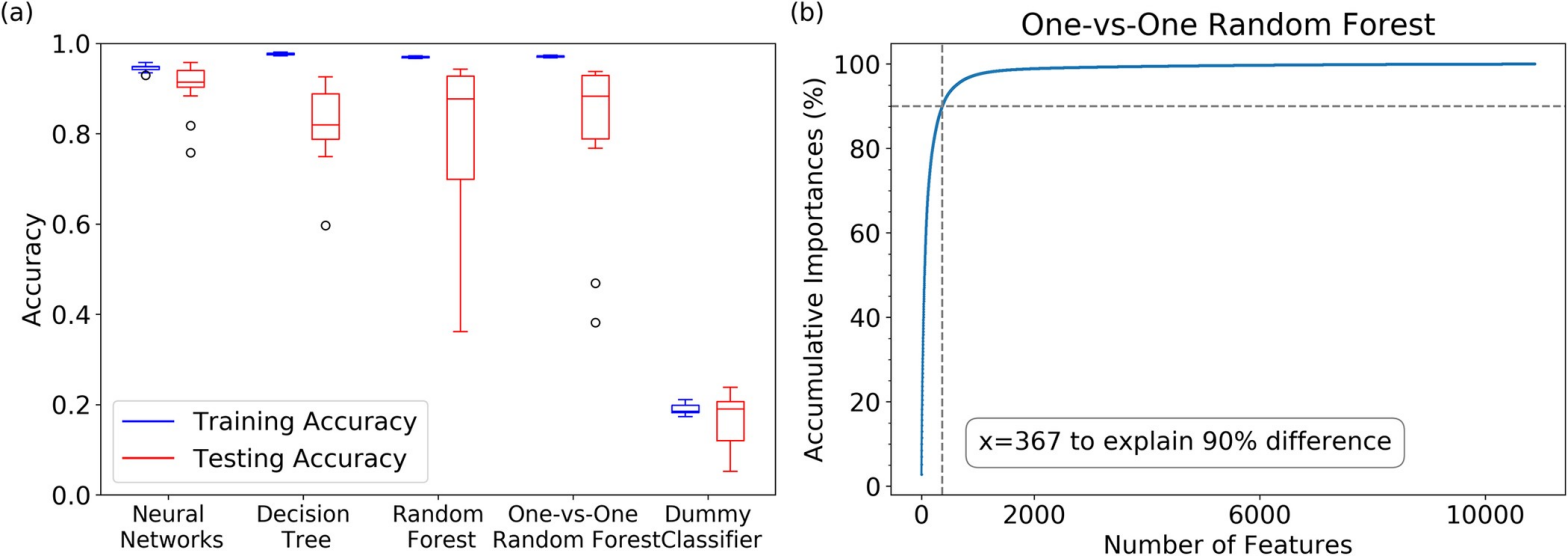
Four machine learning models (artificial neural networks, decision tree, random forest and one-vs-one random forest models) for VVD macrostates: (a) Model accuracy and a dummy classifier as control; (b) One-vs-one random forest classifier accumulating feature importance. One-vs-one random forest is the most effective model with 367 features out of 10,878 features accounting for 90% distinguishability.

The results for optimized machine learning models and a dummy classifier are shown in Fig 7A. Dummy classifier was generated based on random guesses.[43] The training accuracy for neural networks, decision tree, random forest and one-vs-one random forest models are 95.0%, 98.3%, 98.1%, and 99.1%, respectively. The validation accuracy for the artificial neural networks is the highest, having a mean value of 90.1%. Two random forest classifiers and decision tree classifier have relatively lower performances but still significantly higher than the dummy classifier as control. These indicate that the models are able to catch the structural characteristic of each Markov state using the pair-wised Cα distances.

Although artificial neural network model provides the highest classification accuracy, tree-based methods were chosen for further analyses, because these methods could evaluate the contribution from each pair-wised Cα distance. Especially, one-vs-one random forest was applied to compute the feature importance for any two different states pair by performing a random forest classification just between these two states. Therefore, for any two different macrostates, one distinct random forest classifier was built. The combination of 28 basic random forest classifiers, which were calculated as N*(N-1)/2 for eight (*N = 8*) macrostates, were constructed for pair-wised macrostates classification. Overall, this method provides a very effective model, in which 367 features out of 10,878 features account for 90% distinguishability (Fig 7B).

**Fig 8:**
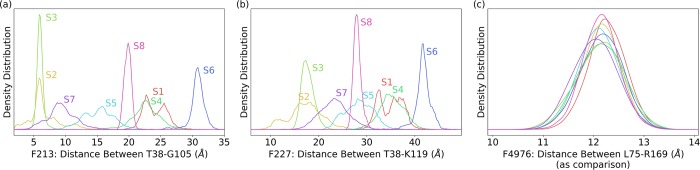
Distributions of selected features (pair-wised Cα distances) in different states. (a) Distribution of Feature 213 as T38/G105 distance in all eight macrostates. This is the top one with overall 2.83% importance. (b) Feature 227 as T38/K119 distance in all eight macrostates. This is the second most important feature with overall 2.38% importance. (c) Feature 4976 L75-R169 with overall 0.0% importance. This unimportant feature has the similar distribution in all eight macrostates.

Top five ranked features computed by the one-vs-one random forest classifier are listed in Table 1. The overall importance of features was calculated by the average of the 28 selected random forest classifiers feature importance. The Cα distances between T38 and G105 is identified as the top feature with the averaged importance as 2.83%. Specifically, it has 4.28%, 4.03%, 4.00% and 4.00% feature importance in distinguishing between States 3 and 5, States 1 and 7, States 5 and 6, States 2 and 6, respectively. The distributions in eight macrostates of top two features are plotted in Fig 8A and 8B, respectively. The most states distributions are well separated based on these two top features. States 2, 3, and 7, which are regarded as ‘dark state’ regions, have shorter distance distributions in both T38-G105, and T38-K119 pairs. States 5, 8, 1 and 4, which are regarded as ‘light state’ regions, have much longer distance distributions than the ‘dark state’ regions. The ‘hidden’ State 6 has the largest distance in both features. For comparison purpose, one of the lowest importance (0.00%) features as feature 4976 between L75 and R169 is also plotted (Fig 8C). For the low ranked features, the distributions for all macrostate are very similar, indicating that those distances are not affected by covalent bond formation as intrinsic allosteric effect.

Comparing with decision tree and random forest, one-vs-one random forests model has at least two advantages. One-vs-one random forest model could provide feature weights specifically for any two different states and is unbiased for features. Overall, only 367 out of 10,878 features have more than 90% distinguishability in one-vs-one random forest model (Fig 7B). The feature importance in one-vs-one random forest model could directly represent the distribution differences between any state pairs for a particular distance. Top ranked features in this model have distinctive distributions in different states (Fig 8A and 8B), while the low ranked features have indistinguishable distributions in all states (Fig 8C), even though those residues are rather far away from each other. This demonstrates the effectiveness of machine learning methods to select features and residues closely related to allosterically important macrostates of VVD.

Although the above structural differences among macrostates revealed through machine learning analysis are informative, some disadvantages do exist. First, the top features could be correlated with each other as the top two features sharing the same residue T38. Second, it could be misleading if only a limited number of top ranked features are selected for investigation since the feature importance between the top ranked and low ranked features are insignificant. Even for top ranked feature 4 and 5 in Table 1, the differences of importance are less than 0.01%. Third, the residues associated with the top features intend to be far away from each other. It is difficult to differentiate these long-distance distribution differences as either directly being correlated with key residues interactions or the result from accumulation of some function related short-range interactions. In addition, the important short-range interactions would have low feature importance, because their distinguishability may not be as significant as the long distance distributions. Therefore, instead of focusing on the residues associated with the top ranked features, we further developed community analysis with more statistical significance.

### Machine learning-driven community analysis specifies a’α/aβ and eα/fα loops as allosteric molecular switches between dark and light states

**Fig 9:**
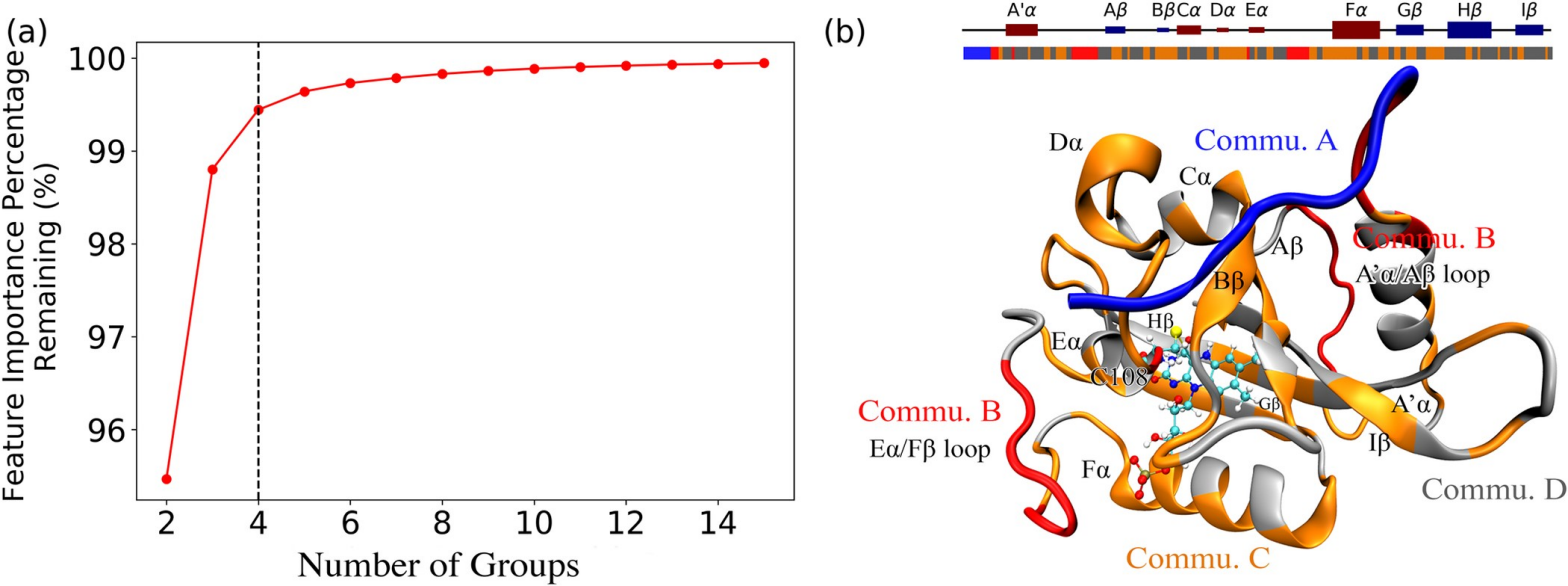
Community analysis of VVD based on one-vs-one random forest model. (a) Total feature importance among ML communities with regard to different number of communities; (b) Four ML communities named Commu. A, Commu. B, Commu. C and Commu. D as blue, red, yellow and grey colors, respectively. The ultimate number of ML communities is determined as four based on the elbow criterion. The feature importance across different ML communities accounts for 99.44% of total importance. The feature importance between Commu. A (which is mainly N-terminus) and the rest of protein accounts for 89.415%. The feature importance between Commu. B and Commu. C as well as D accounts for 9.103% of total feature importance.

Inspired by dynamics network analysis[44], the machine learning based community (referred to as ML community) analysis was developed to divide residues into several groups so that the feature importance for pair-wised Cα distances among groups is maximized, while the feature importance within each group is minimized. The detailed algorithm to construct ML communities is described in the Methodology section. As shown in Fig 9A, with the number of ML communities increasing, the feature importance for pair-wised Cα distances within ML communities increases. Applying an elbow criterion, four ML communities were selected with the total feature importance within each ML community accounting for 0.56% and total feature importance among ML communities accounting for 99.44%. Therefore, the further analysis focuses on the distribution changes among ML communities, neglecting the distribution differences within each ML community, to reveal the overall dynamics associated with ML communities in each configuration. All residues belonging to each ML community are listed in S1 Table in Supporting Information, and plotted in Fig 9(B) named as Commu. A, B, C and D.

Comparing with the secondary structures shown in Fig 9B, Commu. A includes N-terminus from H37 to G43. Commu. B includes the loops in A’α/Aβ and Eα/Fα. It should also be noted that the residue C108 that is bonded to the cofactor also belongs to the Commu. B. Commu. C and D comprise the majority of VVD. Commu. C includes the majority of Aβ strand, Bβ strand, Dα helix, Fα helix and Gβ strand, and part of cofactor binding sites. Commu. D contains the rest of protein, including A’α helix, Cα helix, Eα helix, Hβ stand, and Iβ strand. The N-terminus and loops are well preserved in Commu. A and B, suggesting specific roles of these two secondary structures.

The accumulated overall feature importance between each ML community pair is listed in Table 2. The correlation between Commu. A (which is mainly N-terminus) and the rest of protein accounts for 89.415% total of feature importance in the one-vs-one random forest classifier. This is not surprising since the N-terminus is the most flexible and distinguishable part between the native dark and light states of VVD. However, it should be noted that after excluding Commu. A, the feature importance between Commu. B and Commu. C as well as D still accounts for 9.103% of total feature importance. This suggests that the position of two loops (A’α/Aβ and Eα/Fα) in Commu. B could play an important role to distinguish each macrostate. Although the Commu. C and D comprise the majority of proteins, the accumulated feature importance between them is less than 1%.

**Table 2:** Accumulated feature importance between each ML community pair.

Features	Commu. A	Commu. B	Commu. C	Commu. D
Commu. A	*0*.*041%*	15.885%	37.085%	36.445%
Commu. B		*0*.*107%*	5.236%	3.867%
Commu. C			*0*.*217%*	0.924%
Commu. D				*0*.*191%*

The machine learning based community analysis provides additional information with regard to the different parts of structures during the simulations. In addition to N-terminus, the motions of Commu. B (loops in A’α/Aβ and Eα/Fα) are also significant in distinguishing between the light and dark states. The relative distinguishability of four ML communities associated with key macrostate pairs are listed in Table 3. Besides the N-terminus, the relative position of Commu. B with Commu. C/D is also important to distinguish between the adjacent macrostates (bold in Table 3) including the transition from State 3 to State 2, from State 2 to State 7, from State 7 to State 5, from State 5 to State 4, and from State 4 to State 1. However, for two non-adjacent macrostates, the position of N-terminus (Commu. A) is determinative to the states as shown in Table 3.

**Table 3:** The changes of Commu. A and Commu. B during transitions between states. (Bolded is state-transitions with large Commu B component).

Adjacent macrostates	A with C and D	B with C and D
**State 3 (crystal dark) → State 2**	**38.35%**	**46.38%**
**State 2 → State 7**	**50.12%**	**30.22%**
State 3 → State 7	92.65%	0.87%
**State 7 → State 5**	**59.85%**	**19.37%**
**State 5 → State 4 (crystal light)**	**71.15%**	**17.60%**
**State 4 → State 1**	**50.38%**	**38.01%**
State 7 → State 4	85.09%	0.32%
State 1 → State 8	94.97%	0.52%
State 1 → State 6	74.41%	3.72%
Non-Adjacent macro-states		
State 2 → State 8	79.71%	0.01%
State 3 → State 4	91.27%	0.01%
State 3 → State 6	81.98%	0.01%
State 3 → State 1	78.13%	0.00%

Based on the above results, we hypothesize that when the photo-induced covalent bond is being formed in the dark state, one possible mechanism for protein going through conformational changes from the dark to light state is that the position of Commu. B changes first and subsequently facilitates the conformational change of N-terminus as Commu. A. This transition sequence may have a higher probability than for N-terminus directly changing to another state as shown in Fig 4(A), as well as in Fig 4(B) and 4(C). Overall, VVD has higher probability to switch to the adjacent macrostate with significant changes in Commu. B and little changes of Commu. A. For example, given a structure as dark state conformation starting from state 3, the most likely route to go to light state conformation in state 4 is State 3 **→** State 2 **→** State 7 **→** State 5 **→** State 4 with the probability as 0.15, 0.23, 0.1 and 0.14 in bonded configuration as shown in Fig 4(C). These transitions are shown as bolded state transitions in Table 3 with the highest Commu. B component. Meanwhile, the probability of state 3 directly changing to state 7 or state 7 directly changing to state 4 is much lower as 0.08 and 0.05 in Fig 4(C), and those transitions have larger Commu. A changes as shown in Table 3. These observations indicate that the transition mechanism from dark state to light state with the highest probability is changing the relative position of Commu. B first, instead of changing N-terminus as Commu. A directly. Meanwhile, as shown in Fig 4(B) and 4(C), the bonded configuration has a higher probability to change from the dark to the light conformation than in the non-bonded configuration. Therefore, we hypothesize that the photo-induced covalent bond increases the flexibility of Commu. B comparing to the non-bonded configurations.

To test this hypothesis, the transition pathway theory (TPT)[45] was employed to generate an ensemble of pathways to verify the transition pathway from state 3 (crystal dark conformation) to state 4 (crystal light conformation). Total of 10,017 pathways were generated, and could be grouped as 111 distinct channels floating from state 3 to state 4. The probability of each channel is proportional of the flux through this channel with reference to all channels flux. [45] Overall, the probability for top 20 channels are listed in the Table 4, with the contribution from these channels accounting for more than 98% of total population.

**Table 4:** The probability of top 20 channels.

Channels	Probability
State 3–7–5–4	33.099%
State 3–7–4	21.809%
**State 3–2–7–5–4**	14.819%
State 3–2–7–4	10.369%
State 3–2–4	4.35%
State 3–2–5–4	3.567%
State 3–7–6–1–4	3.008%
State 3–7–1–4	1.257%
State 3–5–4	0.983%
State 3–2–7–6–1–4	0.857%
State 3–7–6–4	0.694%
State 3–2–7–1–4	0.492%
State 3–2–7–6–4	0.464%
State 3–7–5–1–4	0.443%
State 3–2–7–5–1–4	0.387%
State 3–7–5–6–1–4	0.326%
State 3–2–7–5–8–4	0.307%
State 3–2–8–4	0.281%
State 3–7–5–8–4	0.262%
State 3–2–7–5–8–1–4	0.231%
Total 20 channels	98.004%

**Fig 10:**
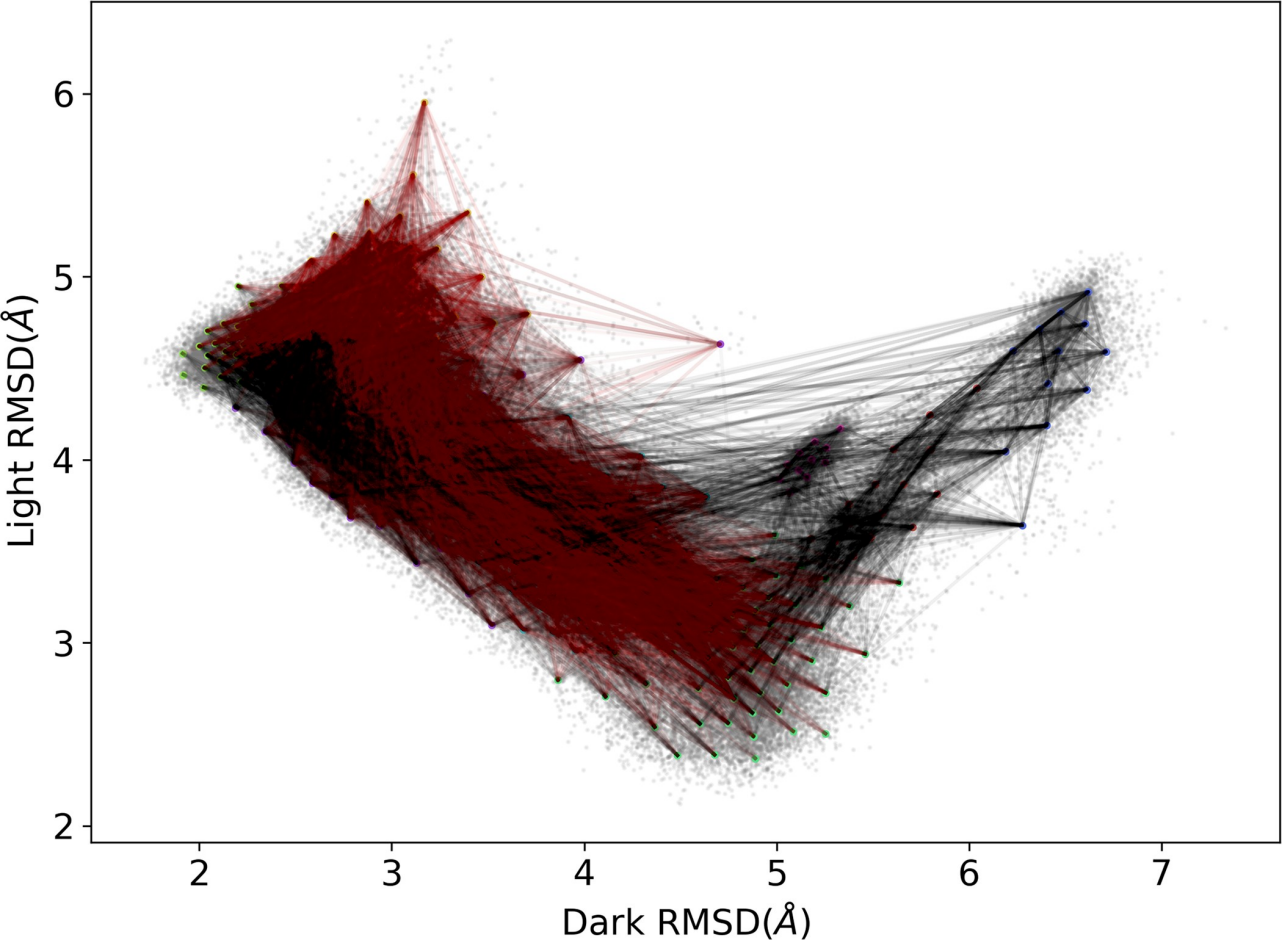
10,017 pathways generated from state 3 to state 4 based on transition pathway theory (TPT). The 3–2–7–5–4 pathway is the third most populated pathways with around 15% contribution and plotted in red. This suggests that the loop regions in Commu. B play an important role during the transition between dark and light states, because the correlations between Commu. B and Commu. C/D contribute significantly to the differentiation between states 3 and 2 as well as states 2 and 7.

Among all 111 channels, the proposed channel 3–2–7–5–4 is the third most populated channels with around 15% contribution (red pathways in Fig 10). Only 3–7–5–4 and 3–7–4 channels have higher contribution. The contribution is significant compared with many other pathways, suggesting the importance of the loop movement during the transition between dark and light states. Besides, the RMSF analysis was also conducted. The results shown in S6 Fig suggest that the photo-induced covalent bond could enhance the fluctuation of A'α/Aβ loop, which may facilitate the transition.

**Fig 11:**
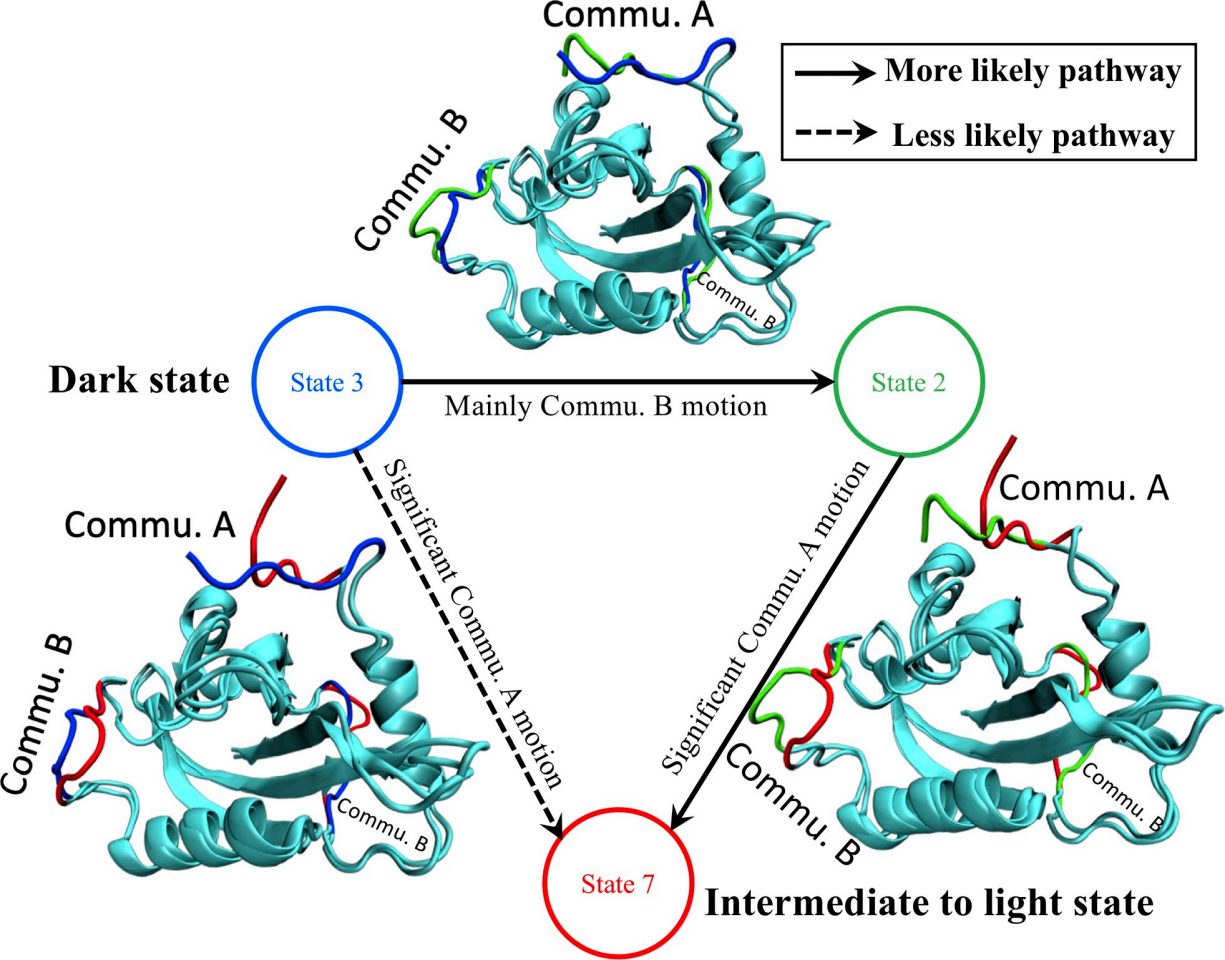
Proposed transition mechanism starting from the dark state of VVD as state 3. Commu. B may function as a switch to be turned on first (State 3 to 2) and lead the conformational change of Commu. A (N-terminus) (State 2 to 7). Commu. A and B are illustrated in blue for State 3, green for State 2, and red for State 7.

To summarize, a general goal of this new community analysis is to minimize the feature weights within each community while maximizing the feature weights among communities. Therefore, we can ignore the internal difference inside each community in different macrostates, and focus on the global differences among communities associated with macrostates. N-terminus standing out as Commu. A is expected, as this is the most distinguishable part between dark and light states. The loops between A’α/Aβ and Eα/Fα standing out as Commu. B provides additional important information to distinguish between the dark and light states. The two loops in Commu. B are far away from each other, but the distance distributions within Commu. B are consistent throughout the dark and light states, with only 0.107% accumulative feature importance. Due to the significant feature importance of Commu. B correlated with the rest of protein, we propose that the two loops in Commu. B mediate the transition from the dark state to the light state (Fig 11). At initial stage of the transition from the dark to the light state, the Commu. B may function as a switch to be turned on first (States 3 **→** 2) before the N-terminus (Commu. A) undergoes significant conformational change to reach an intermediate state (States 2 **→** 7). This route is more likely than the change from State 3 directly to the intermediate state (States 3 **→** 7), in which Commu. A and B undergo the conformational change concomitantly. To further verify the low importance of Commu. C and D during the conformational change, the structural comparison between different macrostates for the proposed pathway is illustrated in S7 Fig and listed in S2 Table. The results clearly suggest that the Commu. C and D do not have significant structural differences among different macrostates, and highlight the conformational changes of Commu. B and A.

## Discussion

### More about community analysis

Some key functional positions have been revealed to control LOV allostery without affecting LOV photocycle kinetics.[4, 46–48] The photo-induced covalent bond between the conserved Cys residue and flavin cofactor initiates conformational changes within N- or C-terminal extensions (Ncap/Ccap) to the LOV core.[49] It was proposed that the conformational change of these N/Ccap elements regulates activity of LOV domains or recruit proteins to Ncap, Ccap or β-sheet surfaces.[4, 50–52]

The results of simulations and machine learning-driven community decomposition allowed to quantify the role of specific regions in allosteric conformational changes and led to major steps of the allosteric mechanism. We found that, in addition to the primary large-scale conformational changes cluster to the N-terminus, the structural changes differentiating N-terminal states are coupled to the rearrangement of two loop regions (A’α/Aβ and Eα/Fα). Therefore, there is a higher probability for covalent bond formation to induce conformational changes in the loop structures first, than to induce reorientation of the N-terminus directly. Notably, although the covalent bond is formed due to the external blue light stimulation, the subsequent conformational changes can be attributed to the existence of the covalent bond without the activation energy dissipations in the protein. Such findings are consistent with recent reports indicating that chemical reduction of the flavin cofactor to form the neutral semiquinone is sufficient to induce a conformational response in VVD, independent of photoexcitation.[53]

Delineating the atomistic details of the allosteric mechanism revealed that focusing on individual residues was insufficient to illustrate a global conformational response. Rather, community analysis presented in this study specified coupling between Commu. B and the N-terminus as Commu. A, where Commu. B is impacted first before significant conformational change occurs at the N-terminus. As a result, residues stabilizing this community could play a central role in switching the conformation of protein from dark to light state. These findings not only reveal significant agreement with experimental observations, but also identify unexpected regions that may play a substantial role in modulating LOV conformational dynamics.

To highlight these findings, we divide Commu. B into three characteristic regions based on experimental data: the molecular swivel (PGG motif residues 42–44), the N-terminal hinge (residues 65–72), and the FAD-binding loop (Eα/Fα loop). Previous experimental studies have identified the first two regions as essential for mediating conformational changes in VVD.[4, 38] Namely, the PGG motif was identified as a molecular swivel that is essential for conformational changes in the N-terminus (N-latch; Commu. A), which distinguishes light- and dark-state conformations. Similarly, mutations in the N-terminal hinge abrogate structural changes. The N-terminal hinge loop between L64-S70 is the last part of N-terminal cap (H37-S70) which is different from other PAS (Per-Arnt-Sim) proteins including photoactive yellow protein (PYP)[54], *Avena sativa* LOV2 domain (AsLOV2) phototropin[51] and *Drosophila* clock protein Period[55]. The mutagenesis studies revealed that the substitution of Cys71, which is the next residues of the identified loop, either enhance conformational changes (C71V) or abrogate the conformational response (C71S) both in vitro and in vivo.[4] Further experimental structural analysis revealed that the hydrogen bond between Asp68 in the identified A’α/Aβ loop with Cys71 could be crucial for N-terminal conformational changes. In addition, the experimental observations show that Pro66, from the identified A’α/Aβ loop, undergoes the largest shifts (2.0Å) in the light state versus dark state[4], which also has agreement with RMSF plot in S6 Fig. Notably, recent studies of other LOV proteins, as well as VVD homologs, identify the N-terminal hinge as a hot spot for evolutionary adaptation, where residues within the core loop facilitate integration of an oxidative stress sensing mechanism into VVD-like proteins by modifying the initial conformational response[38, 56], or aid in differentiation of signaling mechanism by regulating the location of a key evolutionarily selected residue in the adjacent Aβ strand.[57]

### Relation to experimental studies

The synergy between the experimental and theoretical studies not only validates the importance of these structural communities in LOV signal transduction, but also highlights how these communities signal through each other. Here, we show that the photo-induced covalent bond formation first initiates a conformational change in Commu. B, consisting of the swivel and hinge regions. These propagate to the N-latch (Commu. A) to differentiate light- and dark-state conformations. Another question that may arise is if the key conformational changes occur within Commu. A, and B, what are the fundamental function of Commu. C and D. A careful examination of the methodology conducted here and existing experimental studies can shed light on the role of these distinct communities. The current study identified the hinge loop (residues 64–70) as key components of Commu. B, but it did not include residues 71–74 which have been identified as either essential for function (Cys71)[4] or aid in evolutionary adaptation of signaling mechanisms (Ala72 and Ile74)[57]. Rather these residues belong to Commu. C (Cys71) and D (Ala72 and Ile74), as is a key signaling residue in the PAS protein CLOCK (Trp362).[58] None of these residues undergo large conformational changes at the Cα position, rather side chain reorganizations occur due to steric constraints or H-bond changes. Combining these experimental observations indicates that the approach outlined here keenly identifies communities dictating global conformational changes (changes in Cα), but may not include residues near community junctures that enable adaptation in function (Ala72, Ile74) or relay the initial chemical event via a subtle conformational change (Cys71, Ile74). These residues cannot be identified easily by existing computational techniques, because examining every rotamer/H-bond change for its contribution to a global conformational response is not feasible due to the computational time necessary to complete such a task. However, our study indicates these residues likely will reside at the junctures between communities, thus our approach can narrow down candidate residues that may be subtly important for the conformational change, or that are excellent targets for mutagenesis to fine tune signaling mechanisms.

A second unexpected observation of the current studies was the inclusion of the Eα/Fα loop in Commu. B. Currently, the function of the Eα/Fα loop in VVD/LOV signal transduction is largely unknown. It was initially identified as the “FAD insertion loop” due to its presence in fungal proteins VVD and WC1, which were found to bind FAD instead of FMN. Crystal structures of VVD confirmed contacts between the loop and the adenine moiety of FAD[4], however, plant photoreceptors Zeitlupe (ZTL), Flavin-Kelch-Fbox-1 (FKF1) and Leucine-Kelch-Repeat protein 2 (LKP2) all contain a Eα/Fα loop, but selectively bind only FMN.[57] Similarly, experimental studies of VVD homologs in *Trichoderma reesei* and *Botrytis cinerea* confirmed that these proteins bind FMN, despite the presence of the Eα/Fα loop.[56, 59] Thus, the purpose of the Eα/Fα loop remains elusive. Given the unstructured and dynamic nature of the loop, it is particularly challenging to study using traditional experimental approaches.

Here, we identify the Eα/Fα loop as contributing to the initial conformational changes driving rearrangement of the N-terminus, thus the Eα/Fα loop may be a hidden and largely unexplored region to modulate signal transduction in LOV proteins. Indeed, there is some experimental evidence to support such an assertion. Namely, deletions of the Eα/Fα loop were shown to dampen conformational changes in FKF1 that were observed using Small-Angle-X-ray scattering.[60] Furthermore, a recent study identified a mutation in the FKF1 Eα/Fα loop (H105L) that enhanced light-driven activity in designed optogenetic tools.[61] Finally, a possible role of the Eα/Fα loop was also proposed but not confirmed for the VVD homolog in *B*. *cinerea*, where the primary signaling mechanisms were found to diverge from that in VVD.[59] Based on our results, computational approaches to identify how the Eα/Fα loop may modulate signal transduction in LOV proteins, could lead to a new avenue to tune LOV optogenetic tools.

### Concluding remarks

In this work, by using a novel computational framework for dissecting protein allostery, we examined and reconstructed molecular mechanism of Vivid (VVD) protein, which forms a covalent bond between cofactor and a cysteine residue upon blue light activation, and facilitates a large conformational change on N-terminus for circadian signal transduction. By integrating Markov state model, machine learning classification models, and a newly developed community analysis, we accurately reconstructed the equilibrium distributions for bonded and non-bonded configurations, and determined structural differences among these states. A machine learning-based community analysis provided atomistic details of coordinated global motions of functional regions with statistical significance. We systematically verified the impacts of the key local covalent bond upon photo excitation to global motions of VVD, and revealed the importance of A’α/Aβ and Eα/Fα loops in conformational change. The results of this analysis are consistent with the experiments and validated the robustness of the proposed approach in identifying functionally relevant molecular switches of allosteric changes. Overall, this study reveals the detailed mechanism of conformational changes from the dark state to the light state, and the central role of covalent bond in the VVD protein. Our findings also suggested how manipulating these elements with light in LOV proteins can link chemistry with modulation of allosteric changes, thereby providing a path for rational engineering of LOV ontogenetic tools.[1]

## Materials and methods

### Molecular dynamics simulation

The initial structures of dark and light states of VVD were obtained from the Protein Data Bank (PDB)[62] with the ID as 2PD7 and 3RH8, respectively. The dark and light state sequences start from Met36 and His37, respectively. For consistency, residue 36 from the dark state was removed to maintain the same number of residues in both states. Both structures include the flavin adenine dinucleotide (FAD) as cofactor. FAD and flavin monocleotide (FMN) are two types of cofactors commonly existing in the PAS (Per-Arnt-Sim) domain family, and the difference between FMN and FAD comes from the adenosine monophosphate (AMP) moiety. Because FMN and FAD carry similar biological role, the AMP moiety was removed from FAD to construct FMN, and the FMN force field from a previous study was used.[63] Total of four simulation systems were constructed based on crystal dark state structure with or without the photo-induced covalent bond, and crystal light state structure with or without this bond. The crystal dark state structure without the photo-induced covalent bond is referred to as native dark configuration; the crystal light state structure with this bond is referred to as native light configuration. As comparison, the crystal dark state structure with the photo-induced covalent bond is referred to as transient dark configuration, and the crystal light state structure without this bond is referred to as transient light configuration.

Hydrogen atoms were added to the crystal structures, which are subsequently solvated using explicit water model (TIP3P)[64] and neutralized with sodium cations and chloride anions. Initially, 10 nanoseconds (ns) of isothermal-isobaric ensemble (NPT) molecular dynamics (MD) simulations were carried out, and then 1.1 microseconds (μs) of canonical ensemble (NVT) Langevin MD simulation at 300K were conducted. First 100 ns simulations were discarded as equilibration, and the following 1 μs simulation was used in the analysis. Three independent simulations with 1.1 μs length were carried out for each configuration, and total of 12 μs simulations were applied in the analysis. After solvation of the simulation systems, the numbers of TIP3P water molecules added are 7240, 7239, 9430, 9429 for native dark, transient dark, native light, and transient light configurations, respectively. For all simulations, SHAKE method was applied to constrain all bonds associated with hydrogen atoms. Step size of 2 femtosecond (fs) was used and simulation trajectories were saved every 100 picoseconds (ps). Cubic simulation box and periodic boundary condition were applied for all MD simulations. Electrostatic interactions were calculated using particle mesh Ewald (PME) method.[65] All simulations were carried out using CHARMM[66] simulation package version 41b1 with the support of graphics processing unit (GPU) calculations based on OpenMM.[67]

### Markov state model

MSMBuilder[68] was employed to build Markov state model (MSM). To apply MSM, each frame needs to be assigned to a microstate, and transition probability was estimated between different states. To fulfill the “memoryless” assumption underlining MSM, transitions among microstates need to be faster than transitions among macrostates to avoid disguising important kinetic barriers. Therefore, constructing appropriate collective variables (CV) to describe a microstate is critical.[69, 70] Common methods to generate CVs include time structure based independent analysis (t-ICA)[71] and principal component analysis (PCA)[72]. In the current study, the RMSD values calculated with reference to crystal dark and light structures were used as CVs to describe the microstates. 30 ns were chosen as the lag time, and eight macrostates were chosen based on the ‘gaps’ in the implied timescale plot. Perron-cluster cluster analysis (PCCA)[73] implemented in MSMBuilder[68] was applied to cluster the microstate into the macrostates. All the equilibrium or steady state distribution was estimated from the transition probability among different macrostates. In building the MSM, the hyperparameters in MSMBuilder remained as the default setting, including ergodic cutoff being turned on, the reversibility of transition matrix being enforced using maximum likelihood method (MLE), the prior counts for the transition between states being set as zero, and the sliding window setting being turned on. The MSMBuilder used in current study is version 3.8.0.

### Machine learning

Supervised machine learning model including artificial neural network and tree based models were used in the current research. A typical artificial neural network model consists of input layer, hidden layer and output layer with a number of nodes connected with each other. The training processes of artificial neural network model is a back propagation processes implemented in scikit-learn as a Python package.[43] The input data are extracted from the featurization results for each saved simulation frame from trajectories. The target label for each date point is the sequential number of each macrostate. In the artificial neural network model, starting with a random weight assigned to each node, each cycle of training is to minimize the total loss regarding with target label using stochastic gradient descent (SGD) algorithm until weight on each node converges to a minimum. The loss function is defined as

$$Loss(\hat{y},y,W)=-yln\hat{y}-(1-y)ln(1-\hat{y})+a{\left\|W\right\|}^{2},$$ (Eq 1)

whereas *y* is the label predict by the model, $\hat{y}$ is the correct label, ‖*W*‖ is the weights on the nodes, and *a* is named as L2 regulation term to regulate the model to avoid overfitting the weights.

Other supervised models applied in the current study are tree-based machine learning models, including Decision Tree[25], Random Forest[26] and One-vs-one Random Forest. The decision tree is a recursive partition algorithm that groups the samples with the same label together. For a given data set *Q*, the algorithm selects the parameter *θ* = (*j*, *t*) consists feature *j* and a threshold *t* to divide the data into two parts *Q*_*left*_ and *Q*_*right*_ as the following:

$$Q_{left}(\theta )=(x,y)|x_{j}\leq t,$$ (Eq 2)

$$Q_{right}(\theta )=(x,y)|x_{j}>t,$$ (Eq 3)

where *x* is the training data, *y* is the training label. The selection of parameter will minimize the total “impurity” as the following

$$Q^{*}={argmin}_{\theta }\;\left(\frac{n_{left}}{N}*H\;(Q_{left}(\theta ))+\frac{n_{right}}{N}*H\;(Q_{right}(\theta ))\right),$$ (Eq 4)

where H() is the impurity measurement function. Common measurements of the impurity for a given dataset include cross-entropy measurement −∑_*k*_ *p*_*k*_*logp*_*k*_ and Gini impurity ∑_*k*_ *p*_*k*_(1 − *p*_*k*_), where *p*_*k*_ represents distribution of certain class within total dataset. The scikit-learn package employed in the current study used the Gini impurity for training purpose. Therefore, the feature importance is calculated as the sum of all Gini impurity decreasing for all nodes based on the particular feature. However, the algorithm implemented in decision tree models is deterministic with the best splitting of input data, which might be biased towards some features and input conditions.[26] To overcome this, random forest model consisting of multiple decision tree models was applied. In random forest model, each tree classifies the input data with different random seeds, and the final prediction is the average of all single decision tree models. The feature importance from random forest has more statistical significance than single decision tree model. One-vs-one random forest model is a further improvement than the random forest model in multi-class classification task. The one-vs-one classifier is a common strategy in the multi-class classification task.[74, 75] Instead of training only one classifier to classify all classes, one classifier was trained specifically for any two classes pair, and the overall prediction model is weighted by the prediction of all classifiers.[75] Although computational costs are higher than the single classifier, the statistical significance of this model is much higher, and overfitting is less likely. In the current study, for the eight metastable states, total 28 random forest classifiers for state pairs among 1 through 8 were trained. Compared with single random forest model, one-vs-one random forest provides not only the overall feature importance, but also feature weights specifically to distinguish any two particular states.

Pairwise distances for alpha carbons (Cα) were used as features to train the supervised machine learning models. Pairwise distances are invariant with regard to translation and rotation motions of whole molecule. MSMbuilder package was employed to extract Cα pairwise distances from the trajectories. All the machine learning models were implemented using scikit-learn package [43] in python. The performance of machine learning model is assessed by the accuracy of classifier, which is defined as the fraction of the number of the correct classified data with reference to the number of whole input data.

### Machine learning based community analysis

Based on the network and community analysis described in the previous studies,[44, 76] focusing on the community of residues rather than single residues could have more statistical significance. In this study, we propose to group residues into communities, so that the impacts of external perturbations on the distribution differences within the same community are minimum. We refer to these communities as machine learning based communities or simply as ML communities. Therefore, the change of protein motion upon perturbation could be characterized as the relative motion among ML communities related to different states. The feature weights calculated by the machine learning models were applied to construct ML communities. The feature weights indicate the distinguishability between the different states distributions for that specific residue distance. Lower feature weights represent that the specific distance distribution is less distinguishable between different states, and vice versa. Therefore, the community analysis is transformed into a local minimum search problem based on machine learning weights. The Kernighan–Lin algorithm in graph partition problem[77] was implemented to search the local minimum value.

The protein can be modeled as an undirected graph with nodes represented by the residues, and edges represented by the pairwise Cα feature weights. The goal of ML community analysis is to partition the protein graph into several communities and maintain that the total feature importance in each community is minimized. To apply Kernighan–Lin algorithm,[77] we assume that there are *n* communities labeled as *C*_*1*_ through *C*_*n*_. The total feature importance for any partition of communities *T* is defined as the total edge inside each community as the following equation.

$$T=\sum_{l}\sum_{i,j\subseteq C_{l}}E_{ij},$$ (Eq 5)

where *i*, *j* are the residues in Community *C*_*l*_, and *E*_*ij*_ is the feature importance between residues *i* and *j*. The internal edges and external edges for node *i* are defined as the following. Assume that node *i* belongs to Community *C*_*m*_, internal edges of node *i*, *In*_*i*_, is defined as the total edge value between each node in *C*_*m*_ with node *i*, and the external edges of node *i*, *Ex*_*i*_, regarding to any other Community C*q* are defined as the total edges of node in C*q* with node *i*.

$${In}_{i}=\sum_{j\subseteq C_{m}}E_{ij},$$ (Eq 6)

$${Ex}_{i,C_{q}}=\sum_{j\subseteq C_{q}}E_{ij}.$$ (Eq 7)

For each iteration in the algorithm, the ML community partitions can be improved by inserting node *i* into other community or swapping node *i* with node *j* from any different community. For inserting node *i* into community *C*_*q*_, the benefit of total edge in communities is calculated as

$$T_{new}-T_{old}={Ex}_{i,C_{k}}-{In}_{i}.$$ (Eq 8)

For swapping node *i* from community *C*_*m*_ and node *j* from community *C*_*k*,_ the benefit of total edge in communities is calculated as

$$T_{new}-T_{old}=({Ex}_{i,C_{k}}+{Ex}_{j,C_{m}})-({In}_{i}+{In}_{j})-{2*E}_{ij}.$$ (Eq 9)

After defining insertion and swapping operations, the ML community construction algorithm is described as the following:

1. The ML communities are first initialized with random partitions.
2. For each node, the benefits of moving into another ML community are calculated to identify the insertion operation with the maximum benefit.
3. For any pairs of nodes from different ML communities, the benefits of swapping those two nodes are calculated to identify the swapping operation with the maximum benefit.
4. Either swapping or insertion operation with a higher benefit is chosen.
5. For the new community configuration, steps 2 through 4 are repeated until the benefits of insertion or swapping are less than 0.
6. The ML community configuration is final when any insertion or swapping operations will increase the total internal edges within each ML community.

The above algorithm can only reach a local minimum as final solution. Some algorithms like Simulated Annealing[78] could improve the searching for global minimum. In the current study, we repeat 10,000 times with different random starting configurations, and the lowest value was chosen as the final solution.

### Root-Mean-Square Deviation (RMSD) and Fluctuation (RMSF)

The conformational change during the MD simulations can be measured by RMSD regard to a reference structure. For a molecular structure represented by Cartesian coordinate, the RMSD is defined as the following:

$$RMSD=\sqrt{\frac{\sum_{i=1}^{N}{\left(r_{i}^{0}-Ur_{i}\right)}^{2}}{N}}.$$ (Eq 10)

The Cartesian coordinate vector $r_{i}^{0}$ is for the *i*^th^ atom in the reference structure, *r*_*i*_ is the *i*^th^ atom in a given structure. ***U*** is the rotation matrix to superimpose the given structure with the reference structure. *N* is the total number of atoms in the structure. For each simulation, the RMSD values with reference to the crystal dark and light structures were calculated to quantify the sampling following a previous study.[36]

Similarly, the fluctuation of atoms during MD simulation with reference to the averaged structure can be measured by RMSF. The *RMSF*_*i*_ of atom *i* for a given MD trajectory is defined as

$${RMSF}_{i}=\sqrt{\frac{1}{T}\sum_{j=1}^{T}{\left(v_{i}^{j}-\bar{v_{i}}\right)}^{2}},$$ (Eq 11)

where T is the total number of frames in the given MD trajectory, $v_{i}^{j}$ is the coordinate atom *i* in the frame *j*, and $\bar{v_{i}}$ is the averaged coordinate of atom *i* in the given trajectory.

### Transition path theory

After the MSM is established, the transition path theory (TPT) [45, 79] can be applied to estimate the potential transition path related to the conformational changes. Applying TPT for VVD, the target transition paths should connect an initial state A including the native dark macrostate (state 3) and a target state B including native light marcrostate (state 4). All other states are considered as the intermediate states (I). In TPT, the essential concept is “committor probability”$q_{i}^{+}$, which is defined as the probability from any state *i* to reach the target state B rather than initial state A. By definition, all the microstates *i* belonging to state A have $q_{i}^{+}=0$. Meanwhile, all the microstates *i* belonging to state B have $q_{i}^{+}=1$. The commitor probabilities for any other microstates can be calculated by solving the following linear equation:

$$-q_{i}^{+}+\sum_{k\in I}T_{ik}q_{k}^{+}=-\sum_{k\in B}T_{ik},$$ (Eq 12)

where *T*_*ik*_ is the transition probability from state i to state k. The backward-commitor probability $q_{i}^{-}$ is simply calculated as $q_{i}^{-}=1-q_{i}^{+}$. After the commitor probability is calculated, the effective flux from microstate *i* to *j*, which is determined by the transitions from A to B passing through these states, can be calculated as Eq 13

$$f_{ij}=\pi_{i}q_{i}^{-}T_{ij}q_{j}^{+},$$ (Eq 13)

where *π*_*i*_ is the equilibrium distribution for state *i*. The above definition does not consider the backward flux *f*_*ji*_. Therefore, the net flux from A to B transition at edge *i*, *j* can be calculated as $f_{ij}^{+}=max(0,f_{ij}-f_{ji})$. The net flux $f_{ij}^{+}$ is essentially the fluxes leaving state A and reaching state B. Meanwhile, total flux for the transition from A to B per lag time τ can be calculated as the following

$$F=\sum_{i\in A}\sum_{j\notin A}\pi_{i}T_{ij}q_{j}^{+}.$$ (Eq 14)

The flux from state A to state B can be decomposed into distinct individual pathway *P*_*i*_. The pathway decomposition algorithm implemented in MSMBuilder is Dijkstra algorithm, which searches for the highest flux pathway first, then removes the pathway from net flux matrix by subtracting the flux of the path from every edge in the path, and continues search until all possible pathways are identified.

## Supporting information

S1 Fig Comparison between 2D-RMSD, time-structure independent components analysis (t-ICA), and principal component analysis (PCA) models to construct Markov state model (MSM). Projection and grouping of VVD simulations as microstates on the surfaces of (a) 2D-RMSD, (b) t-ICA and (c) PCA and (d) t-ICA with five selected features, respectively. Scanning of lag time for the estimation of relaxation timescales for (e) 2D-RMSD, (f) t-ICA and (g) PCA, and (h) t-ICA with five selected features, respectively. Microstates grouped in eight macrostates on (i) 2D-RMSD, (j) t-ICA, and (k) PCA and (l) t-ICA with five selected features surfaces, respectively.
(TIF)

S2 Fig Averaged Cα RMSD within each microstate in 2D-RMSD, t-ICA, PCA, and t-ICA with five selected features, respectively. (TIF)

S3 Fig Testing the markovian property using Chapman-Kolmogorov method to compare the probability directly observed in the simulation and the estimated probability using lag time as 30ns. (a) VVD dark state 3; (b) VVD light state 4.
(TIF)

S4 Fig Ensemble distributions based on MD trajectories in (a) non-bonded configurations and (b) bonded configurations.
(TIF)

S5 Fig Convergence test of VVD simulations using RMSD and configurational entropy. (a) RMSD fluctuation along each trajectory; (b) The accumulative configurational entropy along each trajectory. The configurational entropy plot indicates that the simulations are well converged after 600ns samplings.
(TIF)

S6 Fig RMS fluctuation analysis of each residue for bonded and non-bonded configurations. The flexibility of A’α/Aβ loop is enhanced upon formation of photo-induced covalent bond between cofactor and VVD.
(TIF)

S7 Fig The representative structures for macrostates 2, 3, 4, 5, and 7, as important for conversion between the dark and light states of VVD. The structure alignment reveals the significant conformational changes of Commu. A and B among different macrostates, and shows that the Commu. C and D do not have significant conformational differences in these macrostates.
(TIF)

S8 Fig Flowchart summarizing the general analysis procedure presented in this study. (TIF)

S1 Table List of residues in each ML community. (PDF)

S2 Table Structural comparison (RMSD in Å) among different macrostates. Communities C and D are combined for the analysis.
(PDF)

S1 Dataset Python scripts implemented in this study with sample data. (ZIP)
