# Supplementary figures and images for: Allosteric mechanism of the circadian protein Vivid resolved through Markov state model and machine learning analysis
Source: PLoS Comput Biol. 2019 Feb 19;15(2):e1006801. doi: 10.1371/journal.pcbi.1006801 (PMC6396943; doi:10.1371/journal.pcbi.1006801)

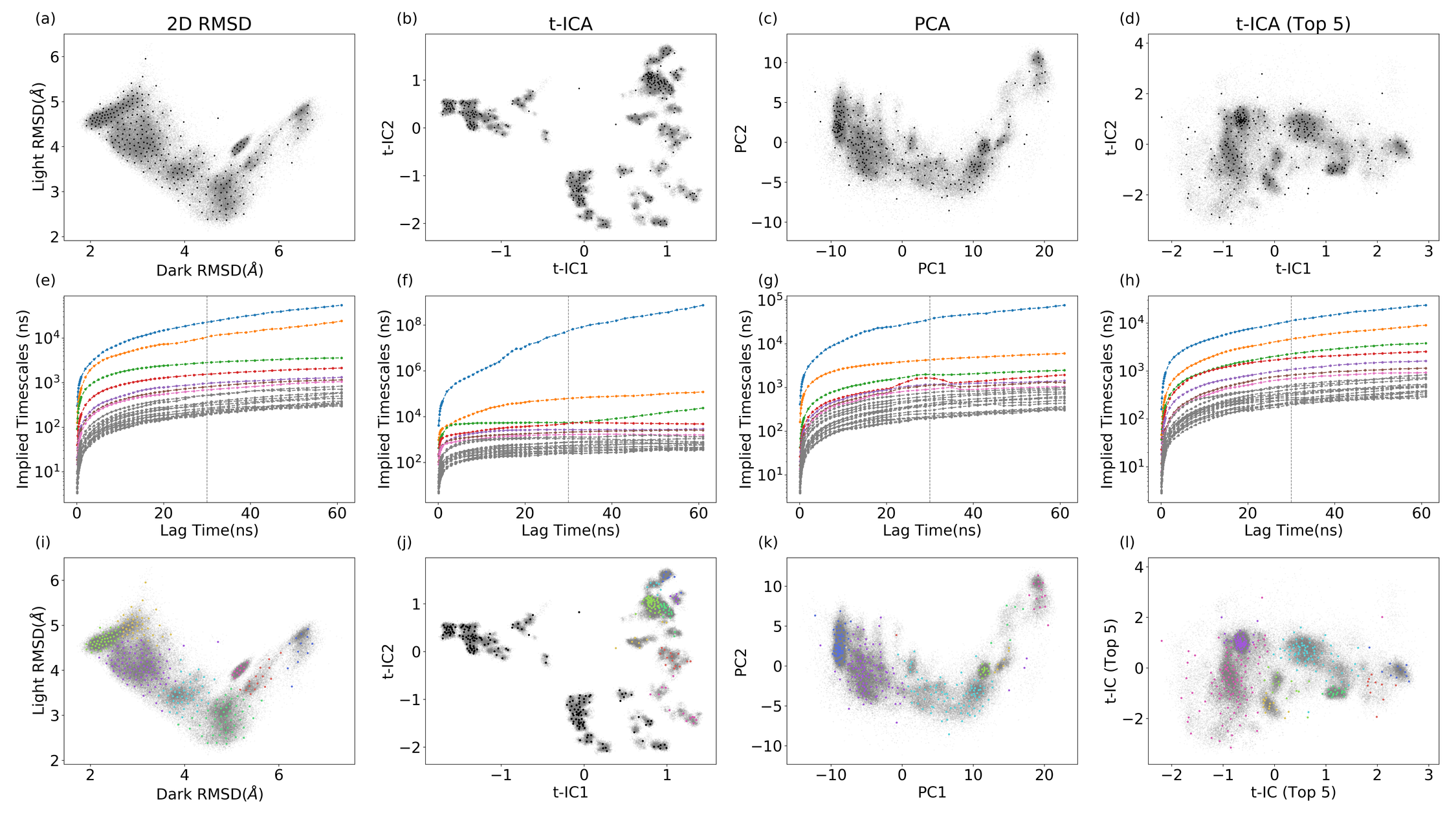

Supplement: S1 Fig — Projection and grouping of VVD simulations as microstates on the surfaces of (a) 2D-RMSD, (b) t-ICA and (c) PCA and (d) t-ICA with five selected features, respectively. Scanning of lag time for the estimation of relaxation timescales for (e) 2D-RMSD, (f) t-ICA and (g) PCA, and (h) t-ICA with five selected features, respectively. Microstates grouped in eight macrostates on (i) 2D-RMSD, (j) t-ICA, and (k) PCA and (l) t-ICA with five selected features surfaces, respectively. (TIF) [file pcbi.1006801.s001.tif]

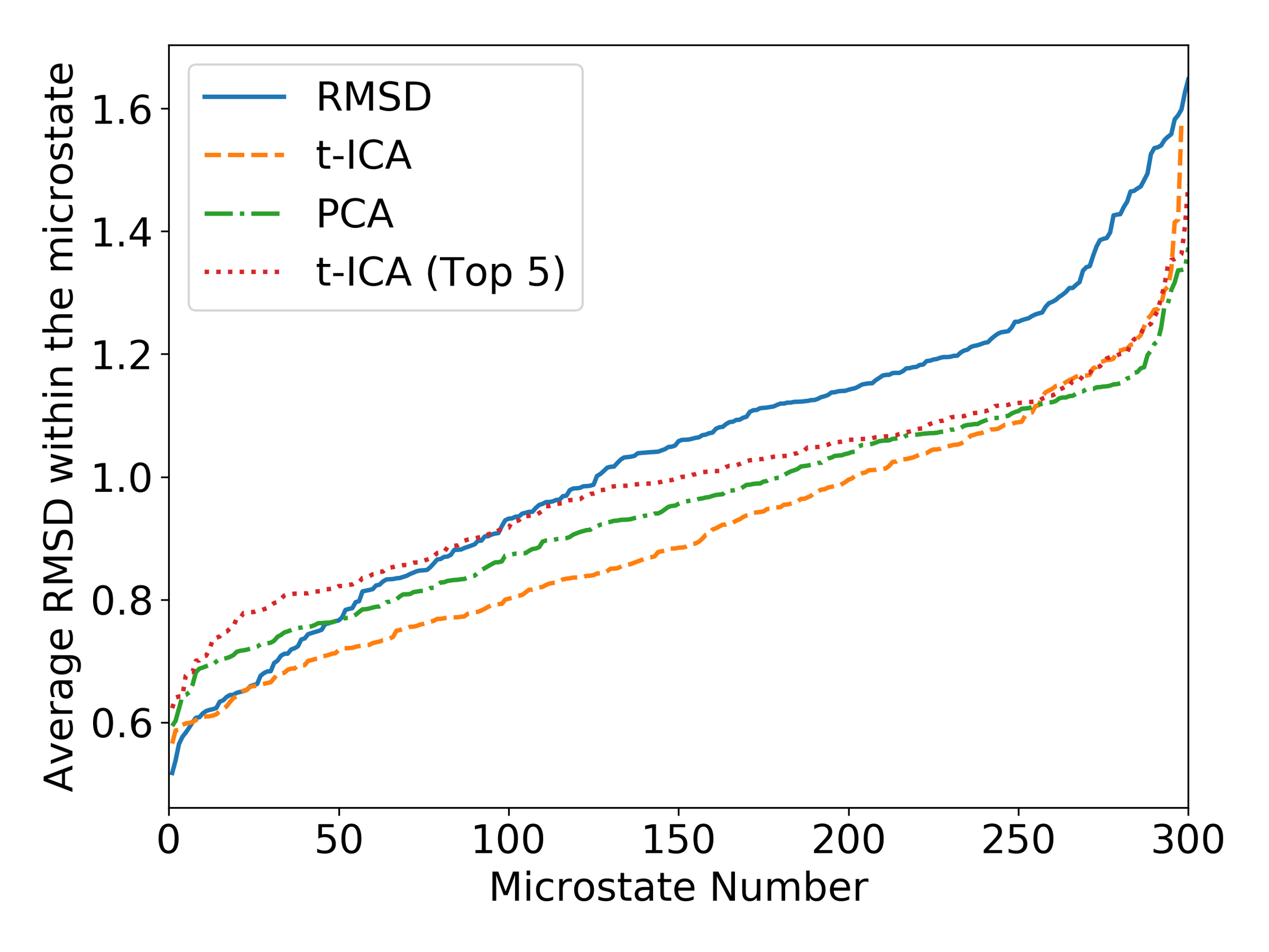

Supplement: S2 Fig — (TIF) [file pcbi.1006801.s002.tif]

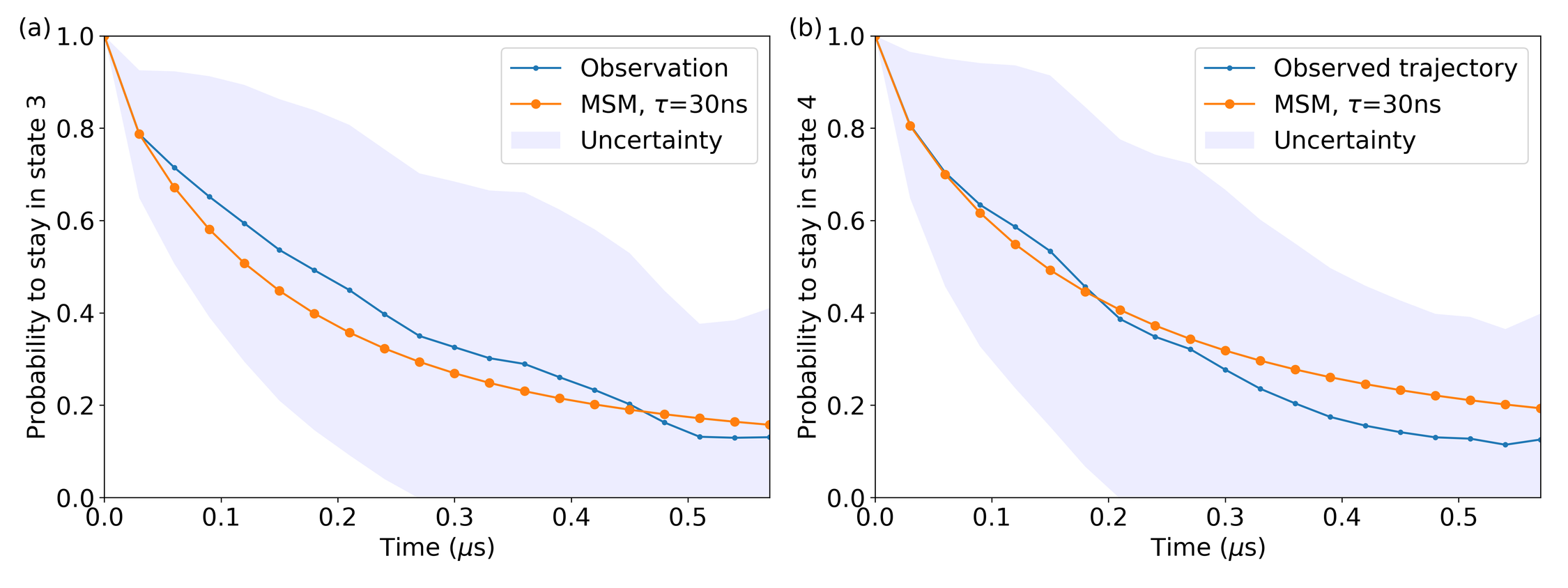

Supplement: S3 Fig — (a) VVD dark state 3; (b) VVD light state 4. (TIF) [file pcbi.1006801.s003.tif]

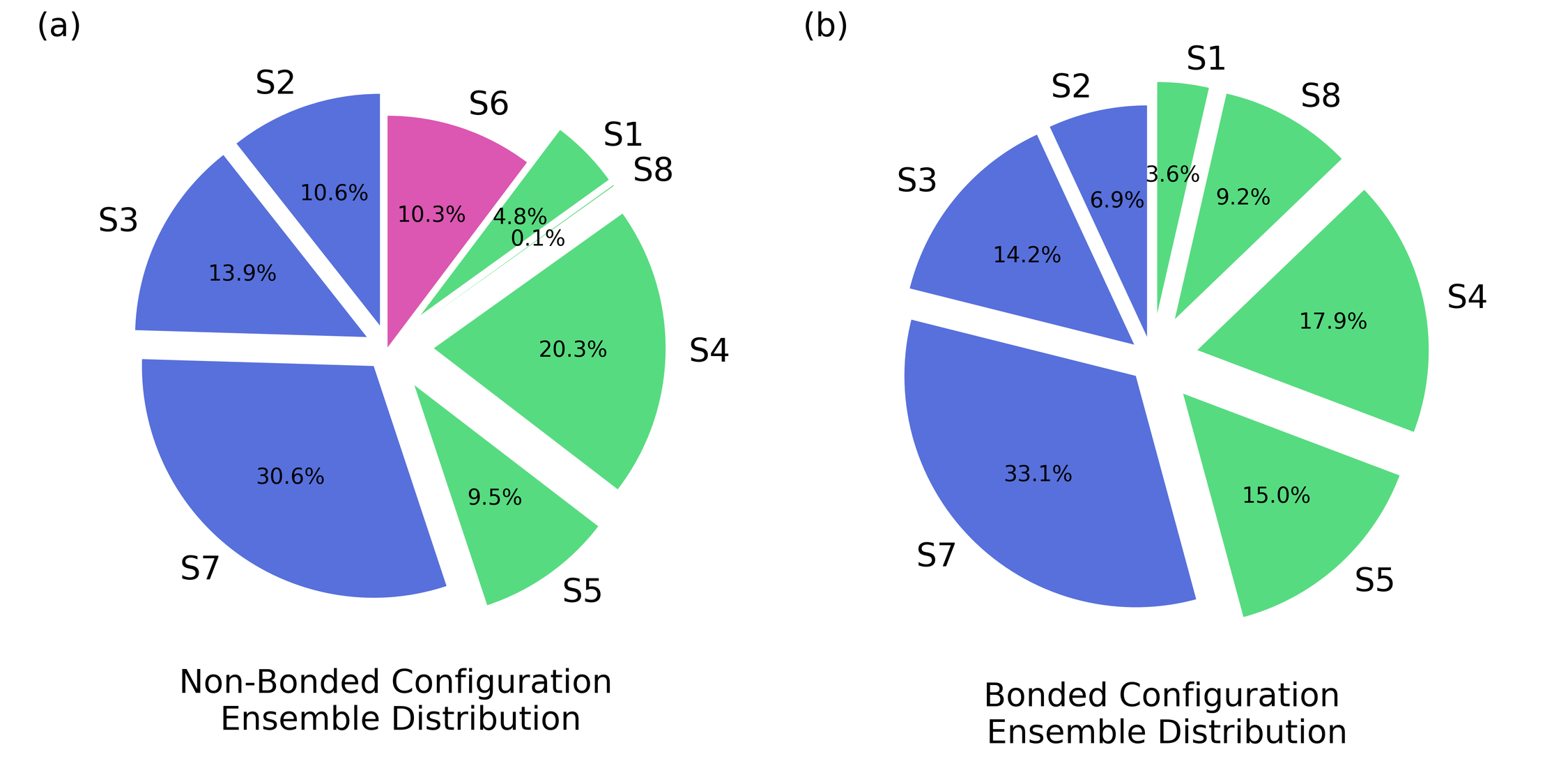

Supplement: S4 Fig — Ensemble distributions based on MD trajectories in (a) non-bonded configurations and (b) bonded configurations. (TIF) [file pcbi.1006801.s004.tif]

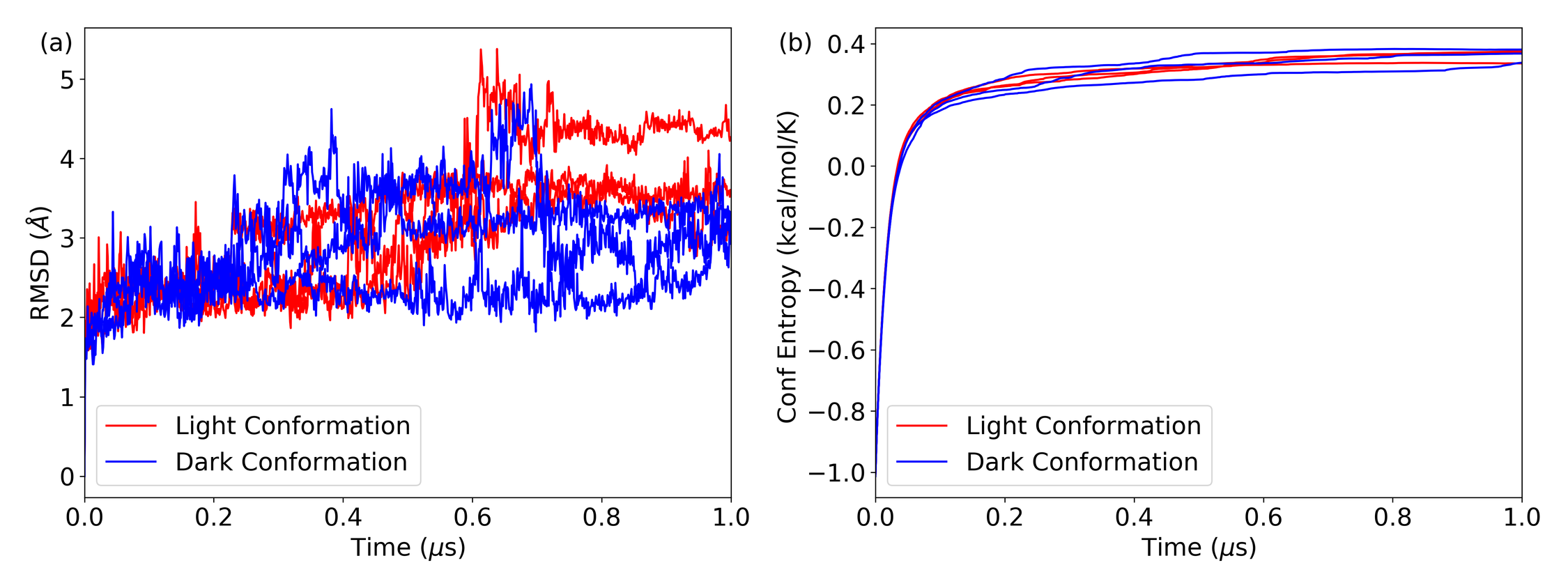

Supplement: S5 Fig — (a) RMSD fluctuation along each trajectory; (b) The accumulative configurational entropy along each trajectory. The configurational entropy plot indicates that the simulations are well converged after 600ns samplings. (TIF) [file pcbi.1006801.s005.tif]

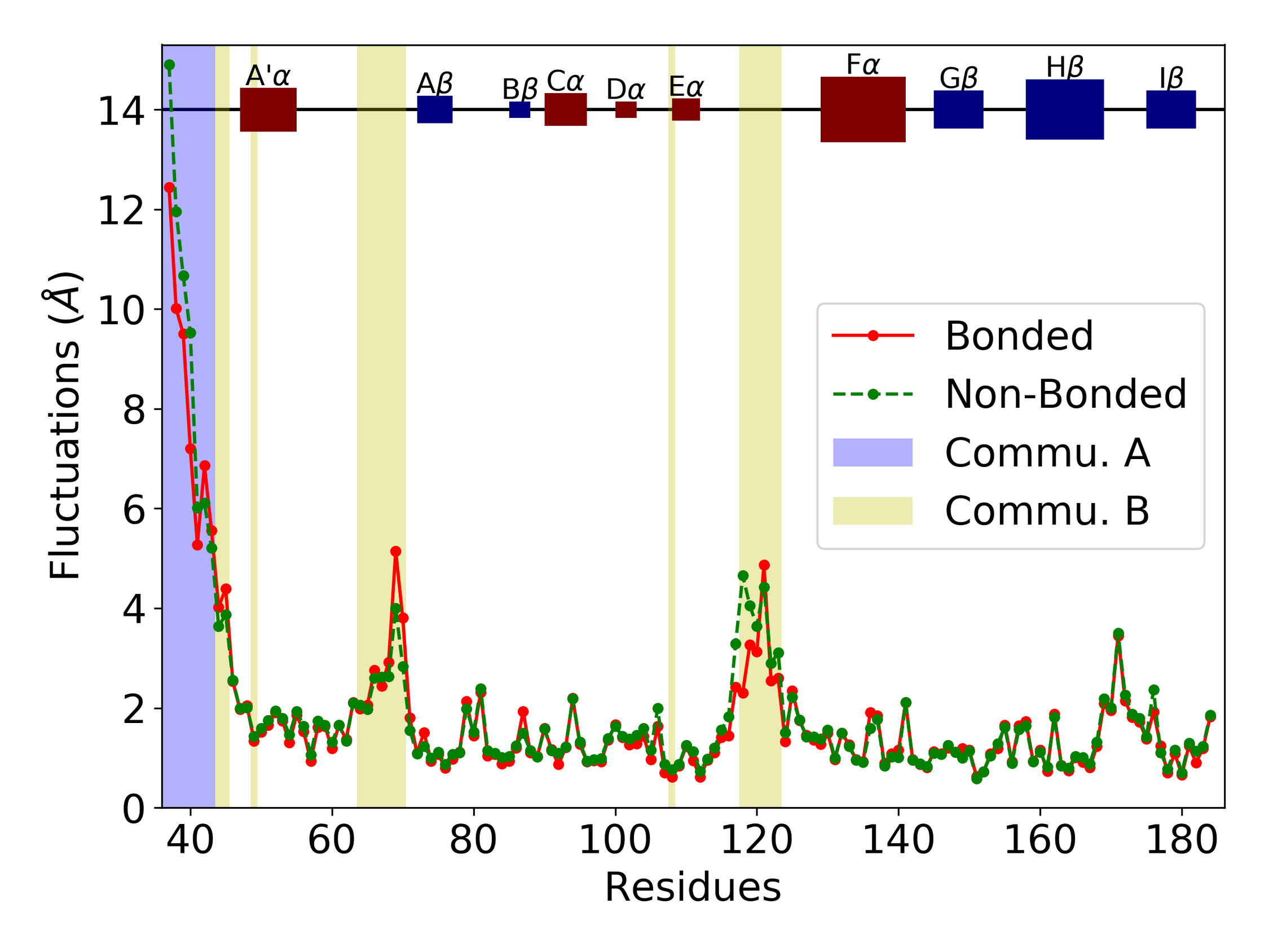

Supplement: S6 Fig — The flexibility of A’α/Aβ loop is enhanced upon formation of photo-induced covalent bond between cofactor and VVD. (TIF) [file pcbi.1006801.s006.tif]

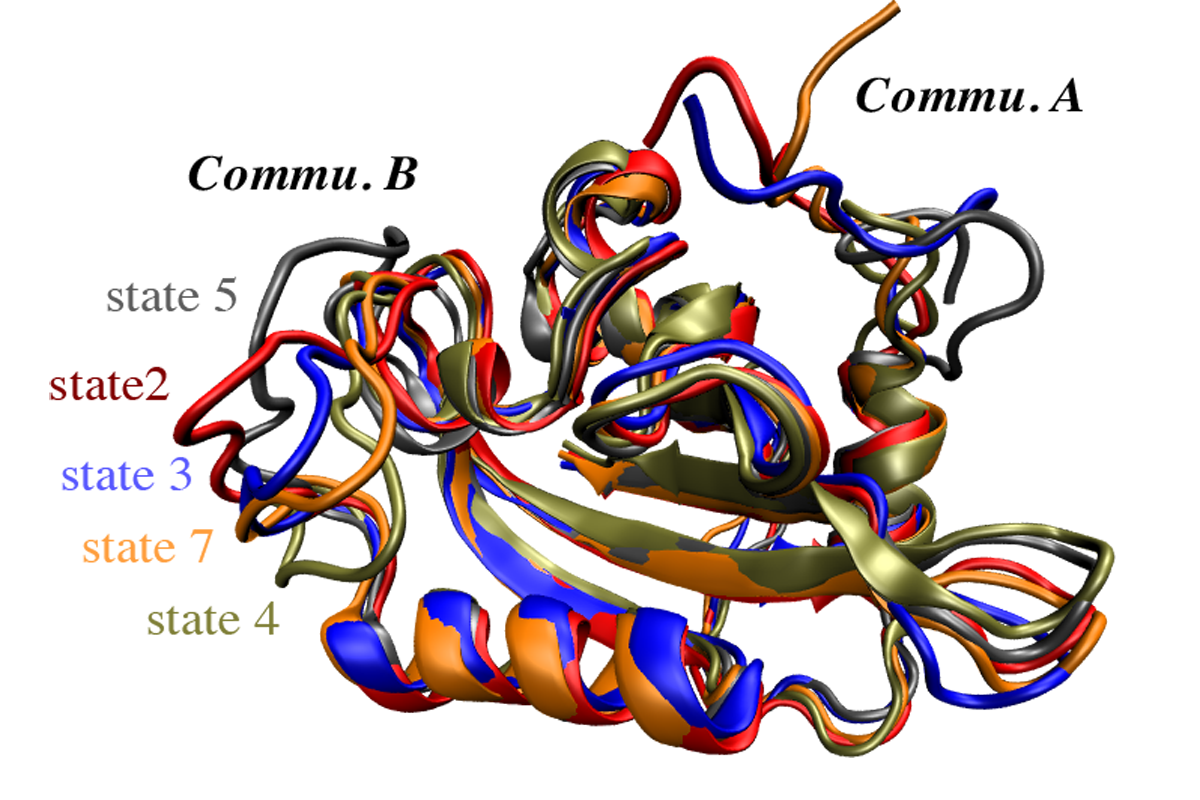

Supplement: S7 Fig — The structure alignment reveals the significant conformational changes of Commu. A and B among different macrostates, and shows that the Commu. C and D do not have significant conformational differences in these macrostates. (TIF) [file pcbi.1006801.s007.tif]

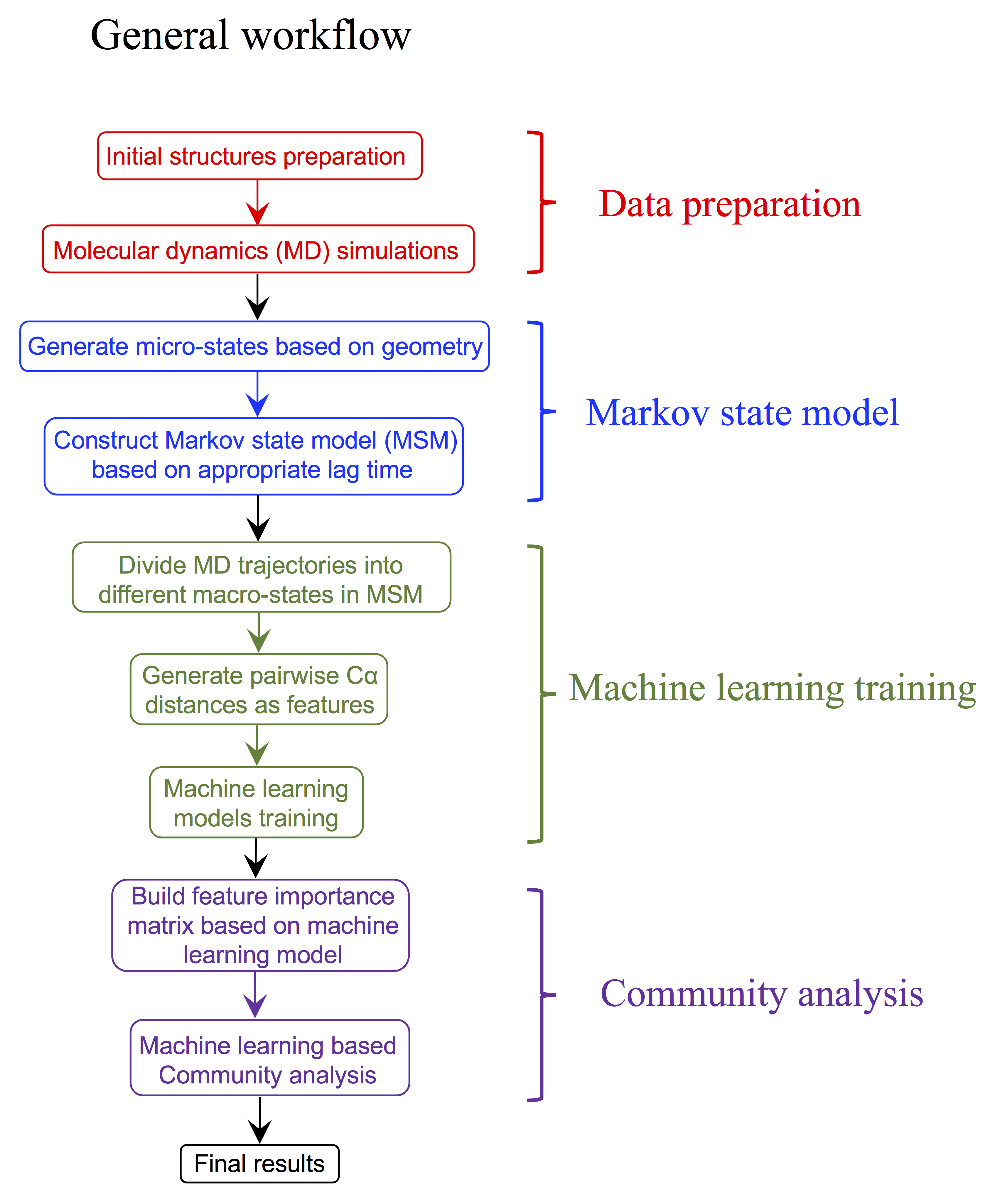

Supplement: S8 Fig — (TIF) [file pcbi.1006801.s008.tif]
