# Supplementary material for: Allosteric mechanism of the circadian protein Vivid resolved through Markov state model and machine learning analysis
Source: PLoS Comput Biol. 2019 Feb 19;15(2):e1006801. doi: 10.1371/journal.pcbi.1006801 (PMC6396943; doi:10.1371/journal.pcbi.1006801)
Supplement: S1 Table — (PDF) [file pcbi.1006801.s009.pdf]

S1 Table. List of residues in each ML community

	Residues
Commu. A	H37, T38, L39, Y40, A41, P42, G43
Commu. B	G44, Y45, G49, L64, G65, P66, V67, D68, T69, S70, C108, V118, K119, P120, K121, S122, T123
Commu. C	D46, Q53, R57, P58, P60, Q61, V62, I74, L75, C76, L78, D82, T83, I85, V86, Y87, A88, S89, E90, L93, Y98, S99, A101, E102, V103, L104, G105, R106, N107, F110, S113, P114, R124, K125, V127, D128, S129, N130, T131, I132, N133, T134, M135, K137, A138, R141, N142, A143, V145, Q146, E148, V149, V150, K153, K154, N155, G156, Q157, L163, T164, I166, E171, E174, R176, Y177, C183
Commu. D	I47, M48, Y50, L51, I52, I54, M55, N56, N59, E63, C71, A72, L73, D77, K79, Q80, K81, P84, A91, F92, Y94, M95, T96, G97, N100, R109, L111, Q112, D115, G116, M117, Y126, R136, I139, D140, E144, V147, N151, F152, R158, F159, V160, N161, F162, M165, P167, V168, R169, D170, T172, G173, Y175, S178, M179, G180, F181, Q182, E184
