# Supplementary material for: Allosteric mechanism of the circadian protein Vivid resolved through Markov state model and machine learning analysis
Source: PLoS Comput Biol. 2019 Feb 19;15(2):e1006801. doi: 10.1371/journal.pcbi.1006801 (PMC6396943; doi:10.1371/journal.pcbi.1006801)
Supplement: S2 Table — Communities C and D are combined for the analysis. (PDF) [file pcbi.1006801.s010.pdf]

S2 Table. Structural comparison (RMSD in Å) among different macrostates. Communities C and D are combined for the analysis.

RMSD (Å)(community A/B/C and D)	State 3	State 2	State 5	State 7	State 4
State 3		2.7/1.4/1.1	4.5/2.3/1.2	3.4/2.1/1.0	2.2/1.5/1.4
State 2	2.7/1.4/1.1		3.3/2.3/1.2	2.6/2.8/0.9	2.3/2.6/1.0
State 5	4.5/2.3/1.2	3.3/2.3/1.2		2.7/3.4/1.1	4.4/2.9/1.2
State 7	3.4/2.1/1.0	2.6/2.8/0.9	2.7/3.4/1.1		2.4/2.2/0.9
State 4	2.2/1.5/1.4	2.3/2.6/1.0	4.4/2.9/1.2	2.4/2.2/0.9	
